# Why isn’t everyone using the thermotolerant vaccine? Preferences for Newcastle disease vaccines by chicken-owning households in Tanzania

**DOI:** 10.1371/journal.pone.0220963

**Published:** 2019-08-15

**Authors:** Zoë A. Campbell, Samuel M. Thumbi, Thomas L. Marsh, Marsha B. Quinlan, Gabriel M. Shirima, Guy H. Palmer

**Affiliations:** 1 Paul G. Allen School for Global Animal Health, Washington State University, Pullman, Washington, United States of America; 2 Nelson Mandela African Institution of Science and Technology (NM-AIST), Arusha, Tanzania; 3 Kenya Medical Research Institute (KEMRI), Kisian, Kenya; 4 School of Economic Sciences, Washington State University, Pullman, Washington, United States of America; 5 Department of Anthropology, Washington State University, Pullman, Washington, United States of America; University of Florida, UNITED STATES

## Abstract

Understanding preferences for veterinary vaccines in low and middle-income countries is important for increasing vaccination coverage against infectious diseases, especially when the consumer is responsible for choosing between similar vaccines. Over-the-counter sales of vaccines without a prescription gives decision-making power to consumers who may value vaccine traits differently from national or international experts and vaccine producers and distributers. We examine consumer preferences for La Sota and I-2 Newcastle disease vaccines in Tanzania to understand why two vaccines co-exist in the market when I-2 is considered technically superior because of its thermotolerance. Household survey and focus group results indicate consumers perceive both vaccines to be effective, use the two vaccines interchangeably when the preferred vaccine is unavailable, and base preferences more on administration style than thermotolerance. Considering the consumers’ perspectives provides a way to increase vaccination coverage by targeting users with a vaccine that fits their preferences.

## Introduction

Consumer preferences play an important role in determining which livestock veterinary inputs are used given the structure of animal health services in low and middle-income countries. In these countries, many consumers of livestock veterinary inputs are smallholder households, defined as small-scale farmers and pastoralists [1]. While comprising about 1.5 billion people, 80% of the world’s farms [1], half the world’s hungry, and three-quarters of Africa’s hungry [2], smallholder farmer households often lack access to veterinary services [3]. The combination of overburdened government veterinary systems, farmer-led demand for vaccines, and limited government regulation means that vaccines and veterinary drugs are usually sold over-the-counter without a veterinary prescription [4]. A study in Kenya showed that, for the poor, access to animal healthcare was mainly through purchasing livestock drugs [5]. Elsewhere, farmers attempt to treat poultry diseases with locally available remedies including human antibiotics and plant-based natural products [6,7]. Veterinary services traditionally provided by governments are being re-examined in the face of changing public and private roles, emphasis on poverty reduction, population growth, and globalization [5,8,9]. Nevertheless, aspects of veterinary decision-making are still left to livestock owners, or consumers, within the context of purchasing veterinary inputs. While it is clear that consumers often make decisions about technically complex products such as vaccines in the absence of professional advice, it is not well understood what preferences or factors drive these decisions or how their choices differ from those with technical expertise.

Thermotolerant vaccine formulations can leave cold-chain conditions for a limited time while retaining potency. Thermotolerance is therefore a highly valued trait from a public health and development perspective because it reduces the dependency on the continuity of the cold-chain system [10,11]. The challenges of maintaining cold-chain systems in low-income tropical and subtropical countries include the transport of vaccines to remote areas, power cuts, outdated refrigerator equipment, poor compliance with cold-chain procedures, and inadvertent freezing during storage or transport [10]. The global eradication of rinderpest, a viral disease of cattle, was, at least in part, dependent on the deployment of a thermotolerant vaccine that allowed field vaccination teams to extend their reach to rural and nomadic communities [12]. Thermotolerant vaccine formulations have been developed for endemic animal diseases, including Newcastle disease affecting chickens [13] and Peste des Petits Ruminants (PPR) affecting sheep and goats [12], with the goal of achieving broader vaccination rates by livestock owners in low-income countries. While the benefits of thermotolerant vaccines are apparent to a variety of stakeholders along the supply chain, it is not well understood how thermotolerance is considered in the decision-making process of final consumers.

The case of Newcastle disease (ND) vaccines in Tanzania provides an example of consumers choosing between two veterinary vaccines, only one of which is thermotolerant. Newcastle disease is an infectious and virulent viral disease that causes high mortality in chickens and is endemic in Africa [13–15]. While there is no treatment, disease can be controlled through vaccination [15]. In Tanzania, two types of live virus lentogenic vaccines are available: La Sota and I-2. La Sota vaccine is older, first introduced to Vietnam in 1968 and Nigeria in 1964 [16,17]. La Sota is imported into Tanzania, distributed by commercial veterinary suppliers, and administered via drinking water. Once the freeze-dried vaccine is reconstituted in water, it must be ingested by chickens within one hour [18]. Most formulations of La Sota are thermolabile [19], leading some researchers to question its ability to be effective in a village setting [13]. Hester Biosciences Limited in partnership with the Global Alliance for Livestock Veterinary Medicines (GALVmed) has developed a thermotolerant ND vaccine using La Sota strain, but at this time, most efforts to introduce it for commercial use have been focused in India [19,20].

Thermotolerant I-2 ND vaccine was developed by the University of Queensland with funding from Australian Centre for International Agricultural Research (ACIAR) and introduced to Tanzania in 1996 [13]. I-2 vaccine can be stored longer in freeze dried form but is typically sold in liquid form in Tanzania [21]. In trials, liquid I-2 vaccine fell below recommended levels of infectivity at two days when stored at 37 °C and at two weeks after storage at variable environmental temperatures (22–29 °C) [13]. I-2 vaccine is produced by government laboratories and is administered via eye drop. Vaccination schedules vary by production style and manufacturer, but most live ND vaccines require re-vaccination at three to four monthly intervals [22]. While there are other ND vaccines with different formulations and/ or different virus strains [15], this study does not attempt to review them all; rather, we consider the two vaccines commercially available in Tanzania at the time of the study and ask what factors influence farmer choice.

This research addresses consumer preferences when choosing between two veterinary vaccines directed against Newcastle disease. We used a mixed methods study to consider how factors such as thermotolerance and administration style affect preferences for ND vaccines available in Tanzania and examine the extent to which consumer preferences contribute to the co-existence of two vaccine types in the market. The results consider factors affecting household decision-making within an animal health care system typical of low-income countries with the goal of improving access to veterinary vaccines.

## Materials and methods

This research was cleared by the Tanzanian Commission for Science and Technology (COSTECH) through permit No. 2018-32-NA-2015-213. The Washington State University Office of Research Assurances found the project exempt from the need for IRB review (#15068).

### Study design

Consumer vaccine preferences are assessed using a cross-sectional household survey, farmer focus groups in six villages, and semi-structured interviews with nine agro-veterinary suppliers (agrovets). Villages were selected using multi-stage sampling approach was used to maximize variation in poultry production practices, access to veterinary services, and household demographics. Arusha, Singida, and Mbeya regions were chosen purposively for their different histories with regards to chicken production. One district in each region was selected randomly, and district governments were consulted to choose two villages in each district. One peri-urban village (< 25 km from urban center) and one rural village (>25 km from urban center) were chosen for a total of six villages. Villages are referred to throughout the paper by their respective region and a 1, indicating peri-urban, or a 2, indicating rural.

Households were randomly selected using a census of heads of households provided by village governments as a sampling frame [23]. A total of 517 households were surveyed about their vaccination practices and preferences [24], but the majority of analyses use a subset of 288 households with experience vaccinating for Newcastle disease. Eligible households had a consenting adult at least 18 years old, currently owned local chickens or had owned them within the past six months, and had previously vaccinated for Newcastle disease. Sample size was not calculated as the research objectives do not involve measuring a population parameter or single proportion; rather the sample size was determined by the number of eligible households with previous vaccination experience. This was deemed sufficient for logistic modeling to identify factors associated with vaccine choice because the data met the guideline of ten outcomes of each type for every predictor [25]. Nine agroveterinary suppliers (agrovets) were identified as accessible to residents of the study villages and an owner or employee participated in a semi-structured interview. Agrovets are for-profit entities present in cities and rural communities that sell agricultural inputs such as fertilizer, seed, pesticides and herbicides as well as animal feed, veterinary drugs, and livestock vaccines. Agrovets are often the immediate upstream link from households in the vaccine supply chain.

### Study sites

Newcastle disease is endemic in Tanzania, where chickens are the most common form of livestock, kept by 86% of livestock-keeping households [13,26,27]. The vast majority of these chickens are indigenous breeds raised in extensive scavenging poultry production systems. The three regions represented in the study span Tanzania’s diversity in geography and climate. Arusha Region in northern Tanzania is the home of ethnic groups such as the Maasai and Arusha that have traditionally focused on raising cattle and small ruminants rather than poultry. Singida Region in central Tanzania has a hot and dry climate, and is known for successful poultry production in part because of easy access to urban consumers by road and rail. Mbeya Region has a cooler and rainier climate, and local governments have hosted some ND vaccination campaigns in the last ten years. The villages have between 1,300 and 3,000 residents and varied in their level of access to veterinary services [24].

### Household survey

Survey questions addressed household socioeconomic status, income, history keeping chickens and other livestock, access to veterinary services, and lastly, knowledge, attitudes, and practices with regards to vaccination. The survey was translated into Swahili, and administered to selected households by pairs of local research assistants, one male and one female. After introductory questions, research assistants asked for the main person in the household responsible for chickens to continue if present. The research focused on indigenous breed chickens and their crosses, hereafter referred to as local chickens. All respondents were read a statement explaining the study and possible consequence of participation. Respondents were informed that participation was voluntary and those choosing to participate provided oral consent. The proposal submitted for IRB review stated that “either written or oral consent will be sought for all participants.” After using written consent forms with a pilot study group, we chose to use oral consent to make participation in the study accessible to respondents with a range of literacy levels. Verbal consent was observed by two research assistants and documented within the electronic survey tool. The survey questions are available in English and Swahili in supplementary materials S1 and S2 Surveys respectively.

### Farmer focus groups

One focus group of six to nine chicken farmers was conducted per village. Households reporting experience with vaccines through recent vaccination (within four months at the time of the survey) were recruited and asked to send a representative family member, preferably the primary person responsible for chickens. By referring to the vaccine type households reported using most recently, we ensured each vaccine type was represented in the focus group discussions by at least two participants. Open-ended questions encouraged lively discussion. Topics included how vaccines work, pros and cons of La Sota versus I-2, and how factors such as price, administration style, flock size, storage and handling requirements and ease of sharing affect vaccine choice. See supplementary materials S1 Text for the focus group questions. Ambient noises during outdoor focus groups, occasional use of local languages in addition to Swahili, and potential for multiple conversations at once contributed to the decision to take notes rather than use audio recordings. Note-takers were trained to include quotations and capture as much of the dialogue as possible.

### Agrovet semi-structured interviews

For each study village, we identified one to two of the nearest agrovets and conducted semi-structured interviews with either the owner or an employee. The goal was to visit all agrovets being accessed by the study villages. All interviews began with questions about price and doses per package of chicken vaccines currently in stock, availability of ND vaccines in the last year, and pros and cons of the two ND vaccine types from their perspective. Open-ended questions followed which allowed for discussion of other factors affecting agrovets when they stock, store, and sell ND vaccines. See supplemental materials S2 Text for the semi-structured interview questions.

### Qualitative analysis

Two to three note-takers took individual notes during focus groups and interviews to minimize response effect and these notes were compiled into a single record [23]. Records were coded for general themes [23] and tallied to identify emergent themes. Since focus groups involved multiple participants, the number of times a theme was re-visited during a single focus group was considered in the selection of the most important themes.

### Statistical analysis

Household survey data were analyzed using logistic regression of vaccine choice conditional on previous vaccination. The outcome variable was the vaccine type households reported using the last time they vaccinated, coded as zero for I-2 vaccine and one for La Sota vaccine. A set of explanatory variables was chosen based on theoretical grounds and variables were tested for independent association with vaccine choice in univariable analyses at 10% significance. Remaining variables were tested in multivariable analyses at 10% significance. Interaction variables were considered if explanatory variables were moderately correlated with a correlation coefficient (> 0.2). The best model presented maximizes goodness-of-fit, minimizes correlation between explanatory variables, and is biased towards retention of explanatory variables of theoretical importance by considering variables at the 10% significance level. We considered alternate models with village as a random effect but the mixed model corresponded with a smaller (worse) log likelihood. By using a fixed effects model, we assume residuals are not clustered by village (See supplementary materials S1–S3 Data and S1 Log for the dataset and selected analyses). Missing data was minimal as the digitized survey required a response for most questions. The largest source of missing data was 8% lost from households that had previously vaccinated but did not report the price they paid for vaccine last time they vaccinated (24/289). Stata uses a complete case analysis so the number of observations was reduced to 264. An additional observation was dropped because it had high leverage and high residual. There was no missing data from any other explanatory variables. All analyses were conducted using Stata 15 [28].

## Results

The quantitative and qualitative results indicate both La Sota and I-2 vaccines are being used in all six study villages, often interchangeably depending on cost and availability. Administration style preferences are more important to vaccine choice than other vaccine qualities such as thermotolerance.

### Quantitative results

The mean flock size for the households with previous vaccination experience was 15 chickens (SE = 1.4) and women were the primary decision-makers for chickens in 63% of households. Farming was the main source of income for 87% of the households. Of households that had previously vaccinated, 78% paid cash to purchase the vaccines. The remainder received free vaccines from non-governmental organizations, friends or family, or through membership in a group. Only 8% of all households reported ever receiving information about chickens from an agrovet, but for the households that vaccinated, the agrovet was the most common source of vaccines. A more detailed description of household characteristics is presented in a previous publication [24].

### Vaccine choice

**Fig 1:**
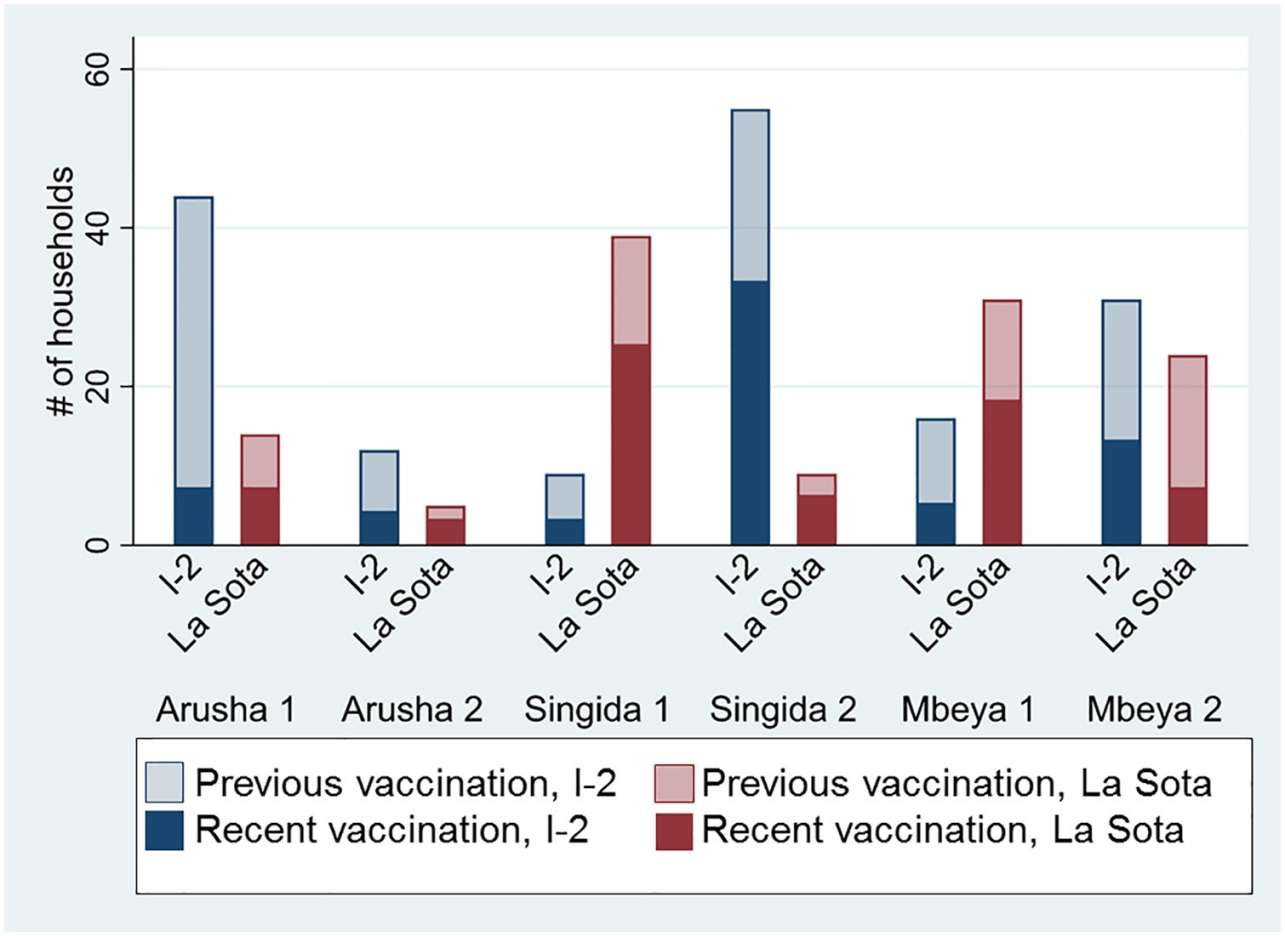
Household vaccine choice between La Sota and I-2 vaccine by village. Dark shading represents vaccine choice of a household that vaccinated within the previous four months and lighter shading the vaccine choice of a household that vaccinated previously but not within the prior four months. (N = 288 for previous vaccination, N = 100 for recent vaccination).

Both vaccines were used in all six study villages as shown in Fig 1. Fifty-eight percent of households reported using I-2 and 42% reported using La Sota the last time they vaccinated (N = 288). For the subgroup of households that had vaccinated recently at the time of the survey, vaccine choice was evenly split between I-2 and La Sota (N = 100), indicating that both vaccines were available in all villages in the four months preceding the survey. Recent vaccination was defined within this study as vaccinating using either La Sota or I-2 vaccine within 120 days, or four months. While vaccination schedules vary by manufacturer, most live vaccines such as I-2 [29] and La Sota require re-vaccination at three to four monthly intervals [22]. We chose 120 days as a threshold in order to identify households that are likely to be in compliance with manufacturer recommendations recognizing that other factors such as presence of chicks or introduction of new chickens to the flock may affect a household’s recommended vaccination schedule.

### Price

Next, we look at price paid per dose of vaccine (total paid last time to vaccinate divided by number of chickens vaccinated) to see how it may affect vaccine choice. Differentiating between sticker price of the vaccine and price paid household is important because vaccines are often packaged with more doses than a household with an average of fifteen chickens needs. The price for La Sota vaccine reported by the nine nearby agrovets ranged from 5,500–7,000 TZS ($2.49-$3.16) for 200–500 chickens and the price for I-2 vaccine ranged from 5,000–7,000 TZS ($2.26-$3.16) for 50–400 chickens. Sharing vaccine with other households or purchasing it as a group can reduce cost.

**Fig 2:**
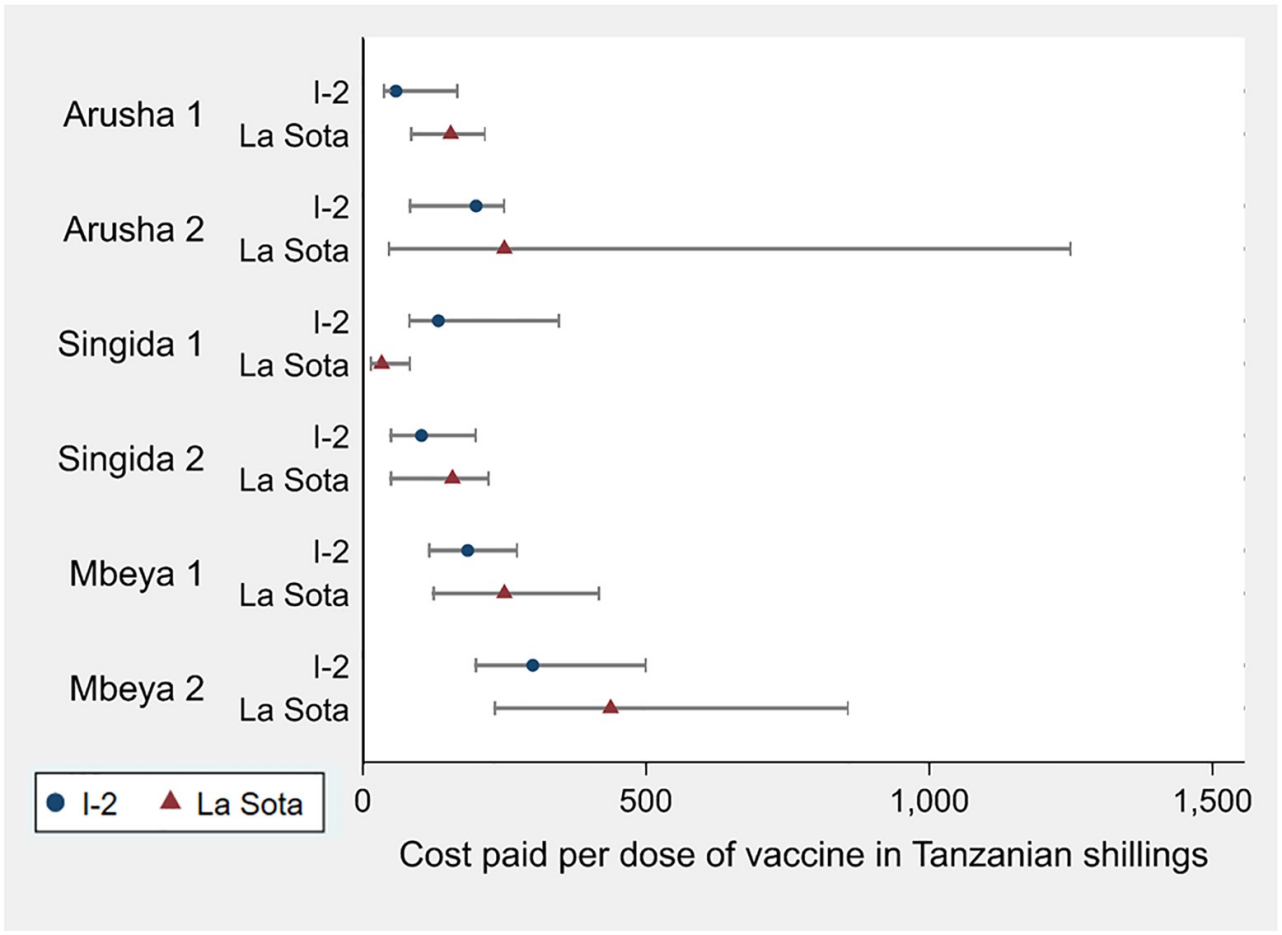
Median price paid per chicken to vaccinate for ND by village and vaccine type in Tanzanian shillings (TZS) with inter-quartile range. (N = 222). $1 USD = 2,213 TZS. Does not include households that received vaccine for free. Only 5 observations for households using La Sota in Arusha 2; all other categories have 9 + observations.

For all households that paid for vaccine, the mean cost per dose was higher for La Sota users (347 TZS or $0.16) than I-2 users (215 TZS or $0.10); t (289) = -2.3, p = 0.02 (using the exchange rate from June 30, 2017: $1 USD = 2,213 Tanzanian shillings (TZS) [30].) Forty-two households reported receiving the vaccine for free, primarily in Arusha 1 where a non-governmental organization provided I-2 vaccine for members of chicken-raising groups. While La Sota vaccine is slightly more expensive overall, there is not a statistically significant difference between the prices paid for La Sota and I-2 vaccines per chicken within any of the villages. Fig 2 shows that the median price paid per dose for La Sota was higher than the price paid for I-2 for all villages except Singida 1. The inter-quartile ranges show overlap between the prices, meaning the least expensive vaccine choice could vary by household.

Determinants of binary vaccine choice between I-2 and La Sota vaccines are presented in the multivariable logistic regression model shown in Table 1.

**Table 1:** Determinants of household choice between I-2 and La Sota vaccines. Multivariable logistic regression model of binary vaccine choice (I-2 = 0, La Sota = 1).

	Odds Ratio	(95% CI)	P-value
Mean price per dose^+^	1.04	(1.02–1.07)	0.001
Singida 1	20.24	(7.82–52.37)	<0.001
Mbeya 1	6.57	(2.95–14.64)	<0.001
Chicken raising group	2.15	(0.99–4.69)	0.054
Vaccine preference: Eye drop			
″Drinking water	3.25	(1.78–5.94)	<0.001
Livestock officer as info source	0.46	(0.20–1.09)	0.077
Intercept term	0.14	(0.07–0.26)	<0.001
Number of observations	263		
Pseudo R^2^	0.27		

(I-2 = 0, La Sota = 1)

^+^Expressed in USD, one unit = $0.01.

All categorical variables are binary, e.g. the reference level for village as Singida 1 is all other study villages.

Price per dose is significant with an odds ratio slightly greater than one indicating that with a $0.01 USD increase in price per mean dose, the household is 4% more likely to be using La Sota vaccine. The model also introduces other explanatory variables important to vaccine choice.

### Administration style and sharing

Preferred administration style is a significant predictor of vaccine choice. Households are more likely to have used a vaccine that matches their preferred administration style, for example I-2 vaccine, if the preferred administration style is eye drop. Interestingly, of the 288 households that reported vaccination, 35% used a vaccine that did not match their preferred administration style. In focus groups, La Sota, administered via drinking water, was identified as easier to share. Households sharing vaccine with others through membership in a chicken-raising group are 2.15 times more likely to be using La Sota, administered via drinking water. Even without a formal group, sharing with other households is common. Of all households that had previous experience vaccinating, 61% shared vaccine with another household the last time they vaccinated (178/292). If the household decision-maker for chickens had completed primary school compared to having no formal education, the household was 2.7 times more likely to share vaccine (p<0.01). If the decision-maker had secondary school education or above, the household was 4.4 times more likely to share (p<0.01).

### Village

All villages were considered as explanatory variables and two remained in the best model. Village serves as a proxy variable for factors outside the household such as varied quality and availability of veterinary services. Singida 1 for example was notable for its active Livestock Officer. The influence of Livestock Officers is apparent from the model; when households report first learning about ND vaccines from a government employed Livestock Officer, they are 54% less likely to have used La Sota vaccine last time they vaccinated. Only 18% of households reported first hearing about ND vaccine from a Livestock Officer (N = 289).

Variables considered but not represented in the final model (P>0.1) include flock size, household income in the previous month, whether the household shared vaccine, knowledge score about Newcastle disease and vaccines over five questions, whether a household member knows someone who vaccinates for Newcastle disease, education level of the primary decision-maker for chickens, region, and an interaction term between education level and sharing vaccine.

### Qualitative results

The qualitative results from farmer focus groups complement the quantitative results, showing that farmers perceive both vaccines to be effective and use them interchangeably depending on availability. Administration style is considered when choosing a vaccine while thermotolerance is not considered important and is in some cases misunderstood. Results from agrovet interviews reveal challenges in the vaccine supply chain that can affect consumer choice.

### Vaccine choice

Forty-one farmers participated across the six focus groups conducted in the different study villages. Forty-six percent of the participants were women. Farmers viewed both I-2 and La Sota vaccines as effective and the majority of the focus group members had experience with both vaccine types. “We have tried them all–La Sota, I-2, traditional medicines”, explained a farmer from Singida region. Participants indicated that the tendency to switch vaccine types was usually a response to changing local availability, as described by a farmer from Mbeya region. “I went to the shop and found the vaccine I wanted was out so I switched.” Four of the nine agrovets addressed the inconsistent availability of vaccines, making it the second most mentioned theme. Other agrovets mentioned that while it might not be possible to stock both vaccine types in multiple package sizes, they could almost always stock ND vaccines of some kind if they chose.

### Price

Discussions of price did not identify a vaccine type that is consistently more affordable; rather focus group participants tended to consider price as a factor for any vaccine. For a chicken owner in Arusha, price was an indicator of vaccine quality. “For those of us who haven’t studied, we look at price. If it is expensive, it’s better. Cheaper, it’s worse.” Others, such as a farmer in Singida, couldn’t help but consider the small size of the purchase of I-2 vaccine—“Six thousand shillings for such a tiny bottle!” Unsurprisingly for a group of recent vaccinators, most agreed that the benefits were worth the price, but a chicken-keeper in Arusha reminded us of the importance of household finances. “Price is a problem for some households! Every rainy season ND comes. Experts tell us and we know, but if I have no money, Newcastle disease will kill my chickens.”

### Administration style and sharing

Despite many having used both vaccines, people had preferences, often related to the vaccine administration style. Table 2 lists emergent vaccine choice themes. La Sota, administered via drinking water, is identified as easy to administer, good for groups, and better for households with fewer chickens. I-2, administered via eye drop, was identified as harder to use but associated with more certainty that all birds have been vaccinated. Concerns that a bird could be “overdosed” by using multiple eye drops were raised in three of the six focus group discussions and by three of the nine agrovets. This misconception is addressed in the discussion.

**Table 2:** Emergent vaccine choice themes from farmer focus groups. All themes mentioned by at least three of the six focus groups are presented in order of frequency of mention.

I-2	La Sota
- Needs cold	+ Easy to administer
- Not available for a period of time	- Uncertainty about whether each chicken received
- Expiration	+ Better for more chickens
- Hard to administer	- Needs cold
+ Certainty each chicken has received	+ Good for groups/ sharing
+ Effective	+ Less expensive
- Easy to overdose*	+ Effective
	+ Better for fewer chickens

*Misconception

Sharing is a way for chicken-owning households to handle cost of vaccines sold in quantities larger than a household may need. “We have to share together. We can’t pay for it [vaccines] all by ourselves,” says a Singida chicken owner. “If you have extra, you have to give it to the neighbors. Our chickens are free range so they can meet and infect each other,” explains another farmer in Mbeya. Sharing is directly related to administration style because half the focus groups mentioned that vaccine administered via drinking water was considered easier to share. Once La Sota is mixed into water, although it only remains viable for about an hour, it is easy to divide into multiple containers to share with other households. I-2, in contrast, is difficult to administer without the eyedropper that accompanies the bottle and it can feel risky to divide the small overall volume.

### Thermotolerance

Four out of six focus groups mentioned “needing cold” for both I-2 and La Sota vaccines. No focus groups mentioned any difference in thermotolerance between vaccines. In fact, “needing cold” was mentioned more times within the focus groups for I-2, perhaps because it is often sold with ice or cold water from the agrovet, in contrast with La Sota which is often pre-mixed with water when sold or distributed. As a chicken owner from Singida explains, “None of the vaccines can just be set on the table. If you get to the shop, you have to explain where you are coming from. Sometimes you will get ice.” Thermotolerance was never mentioned as a criterion for choosing a vaccine type. Instead, farmers consider expiration date and effectiveness, whether the vaccine can be observed to prevent disease (each mentioned by five of six focus groups).

### Agrovet challenges

Power cuts or power outages were the most mentioned theme among the agrovets, brought up by five of the nine agrovets. Seven of the nine agrovets had a refrigerator, typically small and designed for home use. Of the six agrovets who had ND vaccines currently in stock, five owned and used a refrigerator designed for domestic use for primary vaccine storage with ice as secondary storage. One agrovet did not own a refrigerator and relied on ice and a Styrofoam cooler as primary vaccine storage. Only one agrovet owned a generator. “If the power cuts, I move the vaccines to the freezer compartment. I also keep 1.5 liter plastic bottles of water frozen which can keep vaccines cold for two days. Beyond that, I call and we order ice from town,” explained an owner of an agrovet shop in Arusha.

## Discussion

La Sota and I-2 vaccines co-exist in the Tanzanian market even though much of the published literature focuses on I-2 [31,32] with the role of La Sota less frequently acknowledged [33]. Twenty years after in-country production started, the I-2 vaccine valued by experts for its thermotolerance continues to compete with La Sota. The end users of the vaccines are chicken-owning households that perceive both vaccine types to be effective and use them interchangeably, as evidenced by their purchasing behavior and focus group commentary. This is supported by results from the same study framework showing that households had high willingness to pay for ND vaccines, indicating that ND vaccines are valued and preferred [34]. In all six study villages, households report accessing both types of vaccine which confirms that the choice of which vaccine to use is often being made at the household level although availability at the agrovet may dictate choice in some cases. Considering vaccine choice from the perspective of end users explains the persistence of La Sota vaccine, even though it is not thermotolerant, within the market in Tanzania.

### Income and price

Income and price have a predictable effect on consumer behavior, so we discuss their contribution to vaccine choice between I-2 and La Sota. Income in the previous month from both on and off-farm activities was not significant to the model of vaccine choice (P>0.1). Income was also not significantly correlated with a household’s awareness of ND vaccines or previous or recent adoption, however quality of the building materials of the home, another indicator of socioeconomic well-being, was correlated with higher likelihood of awareness of ND vaccines and previous vaccination [24]. In a willingness to pay study, on-farm income, which includes sales of crops and livestock, was associated with a higher willingness to pay for ND vaccines but off-farm income was not significant [34]. These data suggest households with varying levels of income can afford ND vaccines and that income is not a driving factor in the decision between vaccine types.

Across the study, households using La Sota paid more per dose, however the relative costs between La Sota and I-2 varied by village. The variation in relative cost is captured both in the prices paid per dose and by the focus groups, half of which identified La Sota as the less expensive option in their area. In the logistic model of vaccine choice, cost per dose is significant, with an odds ratio of 1.04 indicating as mean price per dose increases, households are more likely to be using La Sota when prices are higher. This result may simply reinforce that La Sota vaccines have a slightly higher average price across all study sites. Given that the data are cross-sectional, the cost difference between the two vaccines may be driving consumer choice or the cost difference may be caused by another factor affecting consumers that is reflected in the price.

The prices paid per dose reported in this study are consistently three to four times higher than prices reported in the literature. Fisher et al. reports a government set price of 50 Tanzanian shillings per dose for I-2 and De Bruyn et al. a price of 100 Tanzanian shillings within the context of a research study [13,35] compared to the mean of 215 Tanzanian shillings in this study. Information about prices paid for La Sota vaccine in smallholder production settings is notably absent in the academic literature. The price descrepancies are a reminder that households incur additional costs in part due to their smaller flock sizes and distance from wholesale suppliers. While price and income are not strong determinants of vaccine choice, limitations on cash flow—not having cash on hand within the household at the time of vaccination- and high transaction costs of accessing cash are still likely barriers to vaccination that can be addressed in future research.

### Administration style and sharing

The difference in administration style between the two vaccines is arguably the most identifiable distinguishing feature for the end user. Administration style contributes to preferences due to differences in ease of use, certainty all chickens have been vaccinated, and ability to share vaccine with other households. It is a misconception that using multiple eye drops when administering I-2 vaccine will cause adverse effects [21]. Additionally, horizontal transfer of the vaccine virus amongst chickens housed together means a missed or suboptimally vaccinated chicken is likely, although not guaranteed, to be protected [21,33].

Notably, 35% of households reported using a vaccine last time that did not match their preferred administration style. Using a less preferred administration style could be a function of price or other factors, but the most likely cause according to the focus groups is availability. Most of the focus group participants had experience with both vaccine types and reported it was common to buy the less preferred vaccine if the more preferred vaccine was unavailable. While a cross-sectional survey has limited ability to look at the choices households make over time, the evidence suggests the two vaccine types are easily substituted.

Sharing vaccine is a trend that should be considered in vaccine development and distribution. Knowing someone who vaccinates increases the likelihood of a household previously and recently vaccinating [24], perhaps in part because of economic benefits of sharing vaccine. Fee-for service ND vaccination programs where trained community vaccinators administer vaccines as described by de Bruyn et al. can facilitate sharing of vaccine vials and potentially allow increased access to vaccines for households with small numbers of chickens [35].

### Thermotolerance

The most mentioned trait of I-2 was that it “needs cold”, mentioned multiple times in four of the six focus group discussions. This is not technically a misconception as I-2 does have specific handling and storage requirements, but serves to reiterate that the benefit of thermotolerance is not well understood or valued by end users. Expiration date and quality were the two most mentioned criteria for choosing a vaccine type within the focus groups. A relatively high level of awareness of expiration dates and cold chain requirements is understandable given that most farmers purchase and administer vaccines themselves, however it is important to remember that the focus groups consisted of households that had recently vaccinated at the time of the survey. This knowledge level may not be representative of those households that vaccinate infrequently. In summary, for end users, thermotolerance does not add much perceived value to the vaccine. From the perspective of farmers without access to convenient refrigeration, both vaccines need to be used quickly; neither can be re-used for the household’s next scheduled vaccination.

### Limitations and future research

Our household survey sample was reduced to only households with previous vaccination experience. This requirement limited our sample size, and consequently, our ability to make conclusive statements at the village level, for example, about price differences between the two vaccines. Focusing on the household does not allow us to address influences farther up the vaccine supply chain such as the levels of production and import or profit margins for distributers even though they are likely to affect household choices. Additionally, a cross-sectional survey limits us from seeing trends in I-2 and La Sota use over time. Discrete choice experiments would be an advisable method for future research to understand how consumers value individual attributes alone rather than in pre-existing combinations. Lastly, color-changing temperature monitors have been used with human vaccines [36] and exploring their applicability for livestock vaccines would engage farmers with the idea of thermotolerance and provide agrovets a more reliable way to gauge vaccine efficacy in addition to relying on expiration dates.

### Conclusion

This study demonstrates that consumers and technical experts value vaccine traits such as thermotolerance and administration style differently. End users do not perceive thermotolerance to add value to the vaccine, even though thermotolerance allows vaccines to reach farmers despite unreliable cold chain in rural Tanzania. In an environment in which consumers make decisions with minimal expert influence, consumer preferences have contributed to the continued use of La Sota vaccine despite its lack of thermotolerance. Consumer preferences suggest that both La Sota and I-2 vaccines can be used to meet the goal of controlling disease and programs to increase vaccination may be most effective if the supply chain provides access to both. As new vaccines are developed and introduced, consumer preferences for administration style and ease of sharing should be considered, especially if multiple vaccines will be marketed to consumers simultaneously.

## Supporting information

S1 Survey Household survey questions in English. (DOCX)

S2 Survey Household survey questions in Swahili. (DOCX)

S1 Text Focus group questions. (DOCX)

S2 Text Agrovet semi-structured interview questions. (DOCX)

S1 Data Household survey data. (XLS)

S2 Data Household survey codebook. (XLS)

S3 Data Household survey data. (DTA)

S1 Log Selected statistical analyses. (DO)

## References

[1] Food and Agriculture Organization of the United Nations. Enduring farms: Climate change, smallholders and traditional farming communities. 2012.

[2] Sanchez PA, Swaminathan MS. Cutting world hunger in half. Science. 2005;307[5708]:357–9. 10.1126/science.1109057 15661994

[3] Food and Agriculture Organization of the United Nations Agriculture Department. Improved animal health for poverty reduction and sustainable livelihoods. FAO animal production and health paper Rome; 2002.

[4] World Bank Group. Drug-resistant infections: A threat to our economic future. Washington DC; 2017.

[5] Heffernan C. Consumer preferences and the uptake of animal healthcare by the poor: A case study from Kenya. J Int Dev. 2001;13[7]:847–61.

[6] Guèye EF. Gender aspects in family poultry management systems in developing countries. Worlds Poult Sci J. 2005;61[01]:39–46.

[7] Snively-Martinez A. Family poultry systems on the southern pacific coast of Guatemala: Livelihoods, ethnoveterinary medicine and healthcare decision making. Washington State University; 2017.

[8] Holden S. The economics of the delivery of veterinary services. Rev Sci Tech. 1999;18[2]:425–39. 10472677 10.20506/rst.18.2.1166

[9] de Haan C. The provision of animal health services in a changing world. Rev Sci Tech. 2004;23[1]:15–32. 15200084 10.20506/rst.23.1.1465

[10] Chen D, Kristensen D. Opportunities and challenges of developing thermostable vaccines. Expert Rev Vaccines. 2009;8[5]:547–57. 10.1586/erv.09.20 19397412

[11] Kristensen DD, Lorenson T, Bartholomew K, Villadiego S. Can thermostable vaccines help address cold-chain challenges? Results from stakeholder interviews in six low- and middle-income countries. Vaccine. 2016;34[7]:899–904. 10.1016/j.vaccine.2016.01.001 26778422 PMC4744085

[12] Mariner JC, Gachanja J, Tindih SH, Toye P. A thermostable presentation of the live, attenuated peste des petits ruminants vaccine in use in Africa and Asia. Vaccine. 2017;35[30]:3773–9. 10.1016/j.vaccine.2017.05.040 28566253

[13] Fisher H. Newcastle disease control in Africa. ACIAR impact assessment series. 2014.

[14] Alders R, Inoue S, Katongo J. Prevalence and evaluation of Hitchner B1 and V4 vaccines for the control of Newcastle disease in village chickens in Zambia. Prev Vet Med. 1994;21[2]:125–32.

[15] OIE World Organisation for Animal Health. Newcastle disease [Internet]. Technical disease cards. 2016 [cited 2016 Jan 1]. http://www.oie.int/fileadmin/Home/eng/Animal_Health_in_the_World/docs/pdf/Disease_cards/NEWCASTLE_DISEASE.pdf

[16] Nguyen TD. Poultry production and Newcastle disease in Vietnam In: Spradbrow PB, editor. Newcastle disease in village chickens: Control with thermostable oral vaccines. Kuala Lumpur, Malaysia: Australian Centre for International Agricultural Research; 1992 p. 169–70.

[17] Olabode AO, Lamorde AG, Shidali NN, Chukwuedo AA. Village chickens and Newcastle Disease in Nigeria In: Spradbrow PB, editor. Newcastle disease in village chickens: Control with thermostable oral vaccines. Kuala Lumpur, Malaysia: Australian Centre for International Agricultural Research; 1992 p. 159–60.

[18] Hipra. Live vaccine against Newcastle disease, La Sota strain, in oral freeze-dried tablet [Internet]. Hipraviar. 2018. https://www.hipra.com/portal/en/hipra/animalhealth/products/detail/hipraviar-s

[19] Hester Biosciences Limited. Facilitating mass access of veterinary vaccines & products to scale-up backyard & small holders farming. 2017.

[20] Bessell PR, Kushwaha P, Mosha R, Woolley R, Al-Riyami L, Gammon N. Assessing the impact of a novel strategy for delivering animal health interventions to smallholder farmers. Prev Vet Med. 2017;147[July]:108–16. 29254707 10.1016/j.prevetmed.2017.08.022

[21] Alders RG, Fringe R, Mata B V. Characteristics of the I-2 live thermostable Newcastle disease vaccine produced at INIVE. In: SADC planning workshop on Newcastle Disease control in village chickens. Maputo, Mozambique: ACIAR Proceedings; 2000. p. 97–100.

[22] Alders R, Spradbrow P. Controlling Newcastle Disease in Village Chickens: A Field Manual. Canberra; 2001.

[23] Bernard H. Research methods in anthropology: Qualitative and quantitative approaches. ed. 4. AltaMira; 2006.

[24] Campbell Z, Marsh T, Mpolya E, Thumbi S, Palmer G. Newcastle disease vaccine adoption by smallholder households in Tanzania: Identifying determinants and barriers. PLoS One. 2018;13[10]. 10.1371/journal.pone.0206058 PMC6200240 30356260

[25] Agresti A. An introduction to categorical data analysis. ed. 2 Hoboken, New Jersey: John Wiley & Sons, Inc.; 2007 138 p.

[26] Tanzania Ministry of Agriculture. United Republic Of Tanzania national sample census of agriculture: Livestock sector. 2012;3.

[27] Da Silva M, Desta S, Stapleton J. Development of the chicken sector in the Tanzanian livestock master plan. Tanzania Livestock Master Plan Brief. 2017;7[October]:1–4.

[28] StataCorp. Stata Statistical Software: Release 15. College Station, TX: StataCorp LLC; 2017.

[29] GALVmed. Protect your chickens against deadly Newcastle Disease [Internet]. 2006 [cited 2018 May 9]. https://www.galvmed.org/wp-content/uploads/2015/07/Newcastle-Disease-Vaccination-Brochure-2016.pdf

[30] Pound Sterling Live. Historical rates for the USD/TZS currency conversion [Internet]. 2017 [cited 2018 Apr 2]. Ahttps://www.poundsterlinglive.com/best-exchange-rates/us-dollar-to-tanzanian-shilling-exchange-rate-on-2017-06-30

[31] Msami H. Poultry sector country review: Tanzania. 2007.

[32] Alders R, Bagnol B, Young M. Technically sound and sustainable Newcastle disease control in village chickens: lessons learnt over fifteen years. Worlds Poult Sci J. 2010;66[03]:433–40.

[33] Bello MB, Yusoff K, Ideris A, Hair-Bejo M, Peeters BPH, Omar AR. Diagnostic and vaccination approaches for Newcastle disease virus in poultry: The current and emerging perspectives. Biomed Res Int. 2018; 10.1155/2018/7278459 PMC6098882 30175140

[34] Campbell Z, Otieno L, Shirima G, Marsh T, Palmer G. Drivers of vaccination preferences to protect a low-value livestock resource: Willingness to pay for Newcastle disease vaccines by smallholder households. Vaccine. 2018; 10.1016/j.vaccine.2018.11.058 PMC6290109 30478006

[35] De Bruyn J, Thomson PC, Bagnol B, Maulaga W, Rukambile E, Alders RG. The chicken or the egg? Exploring bidirectional associations between Newcastle disease vaccination and village chicken flock size in rural Tanzania. PLoS One. 2017;12[11]:1–21. 10.1371/journal.pone.0188230 PMC5690622 29145463

[36] World Health Organization. Temperature monitors for vaccines and the cold chain. Geneva; 1999.

