# Supplementary material for: Why isn’t everyone using the thermotolerant vaccine? Preferences for Newcastle disease vaccines by chicken-owning households in Tanzania
Source: PLoS One. 2019 Aug 15;14(8):e0220963. doi: 10.1371/journal.pone.0220963 (PMC6695108; doi:10.1371/journal.pone.0220963)
Supplement: S1 Survey — (DOCX) [file pone.0220963.s001.docx]

*Note: This survey was programmed and administered electronically using Microsoft Visual Studio.*

**Programmer notes in green**

*Instructions for interviewer in italics*

**Introduction**

We would like to introduce our study project to you and answer any questions you may have. We are conducting this study to improve our understanding of how owners of indigenous chickens protect their chickens against Newcastle disease. The goal is to help chicken owners access technology such as vaccines that can improve the health of their chickens.

The project is funded by the Bill and Melinda Gates Foundation, and supported by Washington State University in the United States and Nelson Mandela African Institution of Science and Technology in Arusha, Tanzania.

You are being asked to take part because your community was selected and your opinion is important to the study.

Participation in the study is entirely voluntary and we understand if you do not have time or do not want to take part.

Taking part in the study will take about 45 minutes. You must be at least 18 years of age to participate, and your household must have at least one indigenous chicken OR has owned at least one local chicken within the last six months.

**Consent and introduction to study** *(Interviewer: I have read the introduction of the study to the primary respondent and they have given consent to participate.)*

**Click “yes” to continue**

| **SECTION A: HOUSEHOLD BASIC INFORMATION** (*Interviewer only)* |
| --- |
| **A1** Date (dd/mm/yyyy): |
| (dd/mm/yyyy) |
| **A2** Interviewer identifier |
|  |
| **A3** Region:  Village:  Sub-village: |
|  |

| **A4** Household identifier |
| --- |
|  |
| **A5** Is the household eligible? |
| 1 = Yes **(GO TO A7)**  2 = No |
| **A6** If no, list reason. |
| 1 = Household does not own at least one local chicken currently or within last 6 months  2 = No adult occupier over 18 yrs  3 = Household has only exotic chickens  4 = Denied consent  5 = Other *(Specify)* ****text box***  **(END SURVEY)** |
| **A7** GPS location (Latitude, longitude) |
| (Format: S ## **° ##.#### ’, E ### ° ##.#### ’** for example **S 03° 23.940’** **E 036 ° 47.958’)** |

| **SECTION B: HOUSEHOLD DEMOGRAPHICS** |
| --- |
| **B1** List all people currently living in household at the current time over 15 years old.  *Complete the table below.* |

|  | **Sex^1^** | **Age**  **(yrs)** | **Marital status^2^** | **Role in family^3^** | **Highest level of education reached^4^** |
| --- | --- | --- | --- | --- | --- |
| Person 1 (Respondent) |  |  |  |  |  |
| Person 2 |  |  |  |  |  |
| Please allow additional spaces |  |  |  |  |  |
| ^1^ **1** = Male, **2** = Female  ^2^ **1** = Married, **2 =** Single, **3 =** Widowed, **4 =** Divorced  ^3^ **1** = Household head; Relative to household head: **2** = spouse, **3** = child, **4** = sibling, **5** = parent, **6** = employee,  **7** = Other *(Specify)* ****text box***  ^4^ **1** = No formal education, **2** = Primary school, **3** = Secondary school (Form IV), **4** = Secondary school (Form VI), **5** = Vocational training, **6** = University | | | | | |

| **B2** List all children under 15 years living in the household at current time.  *Complete the table below.*  Response to B2 not required |
| --- |

| **Person** | **Sex^1^** | **Age**  **(yrs)** | **Currently in school? ^2^** |
| --- | --- | --- | --- |
| Child 1 |  |  |  |
| Child 2 |  |  |  |
| Please allow additional spaces |  |  |  |
| ^1^ **1** = Male, **2** = Female  **^2^1** = Yes, **2** = No | | | |

| **B3** Tribe of head of household: | | |
| --- | --- | --- |
| 1 = Arusha  2 = Bena  3 = Chaga  4 = Gogo | 5 = Hehe  6 = Iraqw  7 = Maasai  8 = Nyiramba | 9 = Sangu  10 = Sukuma  11 = Other *(Specify)* ****text box*** |
| **B4** Does the household have a cell phone? | | |
| 1 = Yes  2 = No **(GO TO C1)** | | |
| **B5** Cell number : Response not required | | |
| Format: 9 digits | | |

| **SECTION C: LIVESTOCK ASSETS** |
| --- |
| **C1** Does the household own livestock? |
| 1 = Yes  2 = No **(GO TO D1)** |
| **C2** What types of animals are owned by the household? How many?  *Write 0 if none are owned.* |

Each space for “Total # of animals owned” required except “Other (Specify)”

| **Livestock type** | **Total # of animals** |
| --- | --- |
| Calves (under 12 months) |  |
| Bulls |  |
| Heifers |  |
| Goats |  |
| Sheep |  |
| Donkeys |  |
| Pigs |  |
| Ducks |  |
| Guinea Fowl |  |
| Other *(Specify)* ****text box*** |  |

| **SECTION D: HOUSEHOLD INCOME** | |
| --- | --- |
| **D1**: What is the main income generating activity of the head of household? | |
| 1 = Farming  2 = Raising livestock  3 = Temporary / short term work | 4 = Business  5 = Employed by government or private company  6 = Other *(Specify)* ****text box*** |
| **D2:** Did any members of the household get income from any of the following activities **last month**?  *Read all responses, select yes or no for each activity.* | |
| \|  \| **Activity** \| S \| \| --- \| --- \| --- \| \| Short term job \|  \| \| Salaried employment \|  \| \| Business \|  \| \| Payment for services \|  \| \| Other payment from projects, government, friends (including benefits in kind (e.g. pension aid, subsidies) \|  \| \| Loans from a bank or lending institution \|  \| \| Informal loans (individual lender or friend) \|  \| \| Renting out your land including farmland or renting buildings \|  \| \| Renting animals for traction \|  \| \|  \| **Sale of crops / livestock** \| \| \| Sale of crops \|  \| \| Sale of chickens \|  \| \| Sale of all other livestock \|  \| \| Sale of eggs \|  \| \| Sale of all other animal products (milk, honey, hides) \|  \| \| Other *(Specify)* ****text box*** \|  \| | |

| **D3:** Which best describes the household’s TOTAL income **last month**? Please consider all sources of income. |
| --- |
| **1 =** 0 tsh  **2 =** 1-25,000 tsh  **3 =** 25,001-100,000 tsh  **4 =** 100,001-200,000 tsh  **5 =** 200,001-400,000 tsh  **6 =** 4000,001-700,000 tsh  **7 =** 700,001-1,000,000 tsh  **8 =** Over 1,000,000 tsh  **777 =** Prefers not to answer |

| **D4:** What are the main materials used to construct the house? *(Interviewer observation)* | | | | | | | | |
| --- | --- | --- | --- | --- | --- | --- | --- | --- |
| 1 = Mud, sticks  2 = Brick or cement | | | | | | | | |
| **D5:** What is the main type of material for the **floor** in the house? *(Interviewer observation)* | | | | | | | | |
| 1 = Earth  2 = Cement | | | | | 3 = Tiles  4 = Other *(Specify)* ****text box*** | | | |
| **D6:** What is the main type of material for the **roof** of the house? *(Interviewer observation)* | | | | | | | | |
| 1 = Natural roofing thatch/palm leaf/ grass  2 = Corrugated iron  3 = Plastic tarp | | | | | 4 = Roofing tiles  5 = Other *(Specify)* ****text box*** | | | |
| **D7:** Does the household own the house you live in? | | | | | | | | |
| 1 = Yes  2 = No | | | | | | | | |
| **D8:** How many rooms does your house have? | | | | | | | | |
| 1 = One room  2 = Two rooms | | | | | 3 = Three rooms  4 = More than three rooms | | | |
| **D9:** What type of fuel do you use to cook? *(Select one)* If 1 is selected, go to D10. If not, skip to D12 | | | | | | | | |
| 1 = Firewood  2 = Charcoal **(GO TO D12)**  3 = Kerosene **(GO TO D12)** | | 4 = Gas **(GO TO D12)**  5 = Electricity **(GO TO D12)**  6 = Solar **(GO TO D12)** | | | | | | 8 = Cow dung **(GO TO D12)**  9 = Other *(specify)* ****text box*** **(GO TO D12)** |
| **D10:** Where do you get firewood? | | | | | | | | |
| 1 = Buy **(GO TO D12)**  2 = Collect | | | | | | | | |
| **D11:** How many hours per day per household is spent collecting firewood? | | | | | | | | |
| 1 = Less than one hour  2 = One to three hours  3 = More than three hours | | | | | | | | |
| **D12:** What is your main source of lighting? *(Select one)* | | | | | | | | |
| 1 = Electricity  2 = Kerosene  3 = Solar | | | | | 4 = Battery powered light  5 = Candle  6 = Other *(Specify)* ****text box*** | | | |
| **D13:** Does the household own any land? | | | | | | | | |
| 1 = Yes  2 = No **(GO TO E1)** | | | | | | | | |
| **D14:** What is the size of the land? | | | | | | | | |
| 1 = Less than one acre  2 = One to three acres  3 = More than three acres | | | | | | | | |
|  | | | | | | | | |
| **SECTION E: WATER AND SANITATION** | | | | | | | | |
| **E1:** What is the household’s source of water for household use?  *(Select all that apply)* | | | | | | | | |
| 1 = Spigot (tap water)  2 = Rivers/streams  3 = Rainwater collection | | | 4 = Well  5 = Pond  6 = Spring | | | 7 = Other *(Specify)* ****text box*** | | |
| **E2:** Do you own a water source? (eg. well) | | | | | | | | |
| 1 = Yes  2 = No | | | | | | | | |
| **E3:** How many hours per day is spent collecting water for the entire household? | | | | | | | | |
| 1 = Less than one hour  2 = One to three hours  3 = More than three hours | | | | | | | | |
| **E4:** What type of toilet facility does the household use? | | | | | | | | |
| 1 = Pit latrine  2 = Improved pit latrine | 3 = No facility  4 = Western flush toilet | | | | | | 6 = Other *(Specify)* ****text box*** | |
| \| **SECTION F: TRAVEL** \| \| \| \| --- \| --- \| --- \| \| **F1** How many trips do household members make to an urban area **per month**? \| \| \| \| 1 = None  2 = One to three trips  3 = Four or more trips \| \| \| \| **F2** What type of transport do you mainly use to go to the urban area? *(Select one)* \| \| \| \| 1 = Walking  2 = Bicycle  3 = Motorcycle \| 4 = Local bus  5 = Bajaj  6 = Truck \| 7 = Car  8 = Other *(Specify)* ****text box*** \| | | | | | | | | |
| **SECTION G: FOOD SECURITY & CHILD PROTEIN CONSUMPTION** | | | | | | | | |
| **G1** Which of the following foods does the household produce? (*Select all that apply)* | | | | | | | | |
| 1 = Grains (eg: corn, rice, millet)  2 = Vegetables  3 = Meat | | | | 4 = Milk  5 = Beans, soy or other legumes | | | | |
| **G2** Is chicken meat consumed in the household? | | | | | | | | |
| 1 = Yes  2 = No **(GO TO G5)** | | | | | | | | |
| **G3** How often is chicken meat consumed in the household? | | | | | | | | |
| 1 = Weekly  2 = Monthly | | | | | 3 = Twice a year  4 = Once a year or less frequently | | | |
| **G4** What is the main source of chicken meat consumed in the household? | | | | | | | | |
| 1 = Produced at home  2 = Bought | | | | | | | | |
| **G5** Are chicken eggs consumed by the household? | | | | | | | | |
| 1 = Yes  2 = No **(GO TO G10)** | | | | | | | | |
| **G6** How many eggs were consumed by the household **in the last three days**? | | | | | | | | |
| 1 = None **(GO TO G10)**  2 = One to five eggs | | | | | 3 = Six to ten eggs  4 = More than ten eggs | | | |
| **G7** How many of these eggs were produced at home? | | | | | | | | |
| 1 = All were produced at home **(GO TO G10)**  2 = One to five eggs | | | | | 3 = Six to ten eggs  4 = More than ten eggs | | | |
| **G8** How many of these eggs were purchased? | | | | | | | | |
| 1 = One to five eggs  2 = Six to ten eggs  3 = More than ten eggs | | | | | | | | |
| **G9** What was the average price paid per egg? | | | | | | | | |
| Tsh……. | | | | | | | | |

| **G10** Please consider meals that the children in the household have eaten in the **last 72 hours (three days)**. *Write 0 if the child did not eat the listed food in the last three days.* |
| --- |

Link to demographic chart for children in household so the additional three columns can be added to information already given regarding gender and age of children under 15 yrs.

| Child | Chicken eggs (# of eggs) | Chicken meat (# meals) | Chicken broth (# meals) |
| --- | --- | --- | --- |
|  |  |  |  |
|  |  |  |  |
|  |  |  |  |
|  |  |  |  |
|  |  |  |  |
|  |  |  |  |

| *Interviewer read:* I’m going to read you several statements that people have made about the food situation in their home. For the following statements, please tell me whether the statement was often true, sometimes true, or never true for (you/your household) in the last 12 months—that is, since last (name of current month). |
| --- |
| **G11** The first statement is, “The food that we produced or bought just didn’t last, and we didn’t have a way to get more.” Was that often, sometimes, or never true for (you/your household) in the last 12 months? |
| 1 = Often true  2 = Sometimes true  3 = Never true  999 = Don’t know  777 = Refused |
| **G12** “(I/we) couldn’t afford to eat balanced meals.” Was that often, sometimes, or never true for (you/your household) in the last 12 months? |
| 1 = Often true  2 = Sometimes true  3 = Never true  999 = Don’t know  777 = Refused |
| **G13** In the last 12 months, since last (name of current month), did (you/you or other adults in your household) ever cut the size of your meals or skip meals because there wasn't enough food or money for food? |
| 1 = Yes  2 = No **(GO TO G15)**  999 = Don’t know **(GO TO G16)** |
| **G14** [IF YES ABOVE, ASK] How often did this happen—almost every month, some months but not every month, or in only 1 or 2 months? |
| 1 = Almost every month  2 = Some months but not every month  3 = Only 1 or 2 months  999 = Don’t know |
| **G15** In the last 12 months, did you ever eat less than you felt you should because there wasn't enough food or money for food? |
| 1 = Yes  2 = No  999 = Don’t know |
| **G16** In the last 12 months, were you ever hungry but didn't eat because there wasn't enough food or money for food? |
| 1 = Yes  2 = No  999 = DK |

| **SECTION H: EDUCATION AND HEALTH EXPENDITURES** |
| --- |
| **H1** How much did the household spent in the last **four months** on school fees? |
| Tsh---- |
| **H2** How much did the household spend in the last **four months** on health-related expenses including clinic visits, hospitalizations, medicine and transportation to health care? |
| Tsh---- |

| **SECTION I: CHICKENS** |
| --- |
| **I1** Who is the primary person in this household responsible for making decisions about chickens?  *(If no, ask to speak to most relevant person if available.)* |
| (Allow choice of people from demographic chart, multiple selection possible) |
| **I1a** Is this person available to continue the interview? |
| 1 = Yes  2 = No |
| **I2** How many chicks, hens, and roosters does the household own? *Complete the chart below. For chicks (under three months), estimate to the nearest 5.* |

|  | **# of birds** |
| --- | --- |
| Roosters |  |
| Hens |  |
| Chicks (<3 months) |  |

| **I3** What types of chickens do you have in your flock currently?  *(Select all that apply)* |
| --- |
| 1 = Local chickens  2 = Layers  3 = Broilers |
| **I4** The following questions are about the movement of chickens into and out of your household.  *Please complete for movement of chickens owned by the household* ***in the last month****. List the number of individual animals. Write zero if necessary. For chicks, estimate to the nearest 5.*  Each non-shaded box requires an answer, prompt to write zero if not applicable. |

|  | Roosters | Hens | Chicks (< 3 months) | **TOTAL** |
| --- | --- | --- | --- | --- |
| Hatched |  |  |  |  |
| Bought |  |  |  |  |
| Received as gift |  |  |  |  |
| **Total # animals IN** |  |  |  |  |
|  | | | | |
| Sold |  |  |  |  |
| Given away |  |  |  |  |
| Predators killed |  |  |  |  |
| Stolen |  |  |  |  |
| Died of disease and eaten |  |  |  |  |
| Died of disease and thrown away |  |  |  |  |
| Slaughtered and eaten at home |  |  |  |  |
| **Total # animals OUT** |  |  |  |  |

| **I5** Why do you keep chickens? (*Select all that apply)* | | | |
| --- | --- | --- | --- |
| 1 = Income from selling chickens  2 = Income from selling eggs  3 = Sell to pay for household needs  4 = Resolve problems | | 5 = Food  6 = Guests  7 = Other *(Specify)* ****text box*** | |
| **I6** Select the option that best describes the living conditions of your chickens. | | | |
| 1 = Free range, roost outdoors at night  2 = Free range, brought inside courtyard or structure at night | | 3 = Enclosed at all times, provided feed | |
| **I7** Are the chickens fed any improved foods / supplements? (eg: bone meal, calcium, sunflower milling remains, sardines) | | | |
| 1 = Daily  2 = Weekly | | 3 = Once or twice a month  4 = Not fed improved feed | |
| **I8** For how long have you kept chickens? | | | |
| 1 = Less than one year  2 = One year to five years | | 3 = More than five years | |
| **I9** Who in the household is responsible for the day to day care of the chickens? *(Select one)* | | | |
| 1 = A resident of the household (Link to drop down menu with info from demographic chart to interviewer can choose appropriate person # )  2 = Employee of the household | | | |
| **I10** Who is responsible for deciding if a chicken should be sold or bought? *(Select all that apply)* | | | |
| (Link to drop down menu with info from demographic chart so interviewer can choose appropriate person #. Allow selection of two people maximum. ) | | | |
| **I11** Where do you normally sell eggs? *(Select all that apply)* | | | |
| 1 = Store  2 = Market  3 = French fry vendors  4 = Restaurant | 5 = Roadside  6 = Retailer/ middleman  7 = Walking sales  8 = Individuals | | 9 = Train station  10 = Church/ Mosque  11 = Holiday  12 = Don’t sell eggs  13 = Other *(Specify)* ****text box*** |
| **I12** Where do you normally sell chickens? *(Select all that apply)* | | | |
| 1 = Market  2 = Monthly market  3 = French fry vendors  4 = Restaurant | 5 = Roadside  6 = Retailer/ middleman  7 = Town  8 = Individuals | | 9 = Train station  10 = Church/ Mosque  11 = Holiday  12 = Don’t sell chickens  13 = Other *(Specify)* ****text box*** |
| **I13** How many of your chickens are currently laying eggs? | | | |
| (# of birds) | | | |
| **I14** How many eggs does your flock lay on average per week? | | | |
| (# of eggs) | | | |
| **I15** How many chicken eggs do you sell each week? **If 0, skip to I17** | | | |
| (# of eggs) | | | |
| **I16** What price do you receive for selling per local chicken egg? (tsh) | | | |
| Tsh-- | | | |
| **I17** How many local chickens do you sell per month? (# of chickens) **If 0, skip to I 19** | | | |
| (# of chickens) | | | |
| **I18** What price do you receive for selling one local chicken on average? (tsh) | | | |
| Tsh-- | | | |
| **I19** What factors keep you from increasing the size of your flock?  *(Select all that apply, do not read responses)* | | | |
| 1 = Disease  2 = Selling chickens  3 = Thieves  4 = Predators  5 = Death of chicks | 6 = Limited space  7 = Traditional husbandry  8 = Few eggs laid  9 = Not using vaccines | | 10 = Not following vaccine schedule  11 = Little education  12 = Carelessness  13 = Other *(Specify)* ****text box*** |
| **I20** Are you able to buy young chicks from a hatchery in your area? | | | |
| 1 = Yes  2 = No  999 = Don’t know | | | |
| **I21** Do you feel that raising chickens is a profitable business activity in your area? | | | |
| 1 = Yes  2 = No | | | |
| **I22** Do you belong to a chicken raising group? | | | |
| 1 = Yes  2 = No | | | |

| **SECTION J: VACCINATION SERVICES AND NEWCASTLE DISEASE** | | | |
| --- | --- | --- | --- |
| **History:** | | | |
| **J1** In the past **six months**, have you had any episodes where a third of more of your chickens died? | | | |
| 1 = Yes  2 = No **(GO TO J4)** | | | |
| **J2** What do you think caused the most recent loss?  *(Do not read responses, select one)* | | | |
| 1 = Newcastle disease  2 = Fowl pox  3 = Gumboro/ Infectious Bursal Disease | | 4 = Other *(Specify)* ****text box***  999 = Don’t know | |
| **J3** Did you observe any of the following clinical signs during the most recent episode?  *(Read all responses and select all that apply)* | | | |
| 1 = Coughing  2 = Sneezing  3 = Difficulty in breathing  4 = Nasal discharge | 5 = Diarrhea  6 = Nervous problem i.e. twisting of the neck, depression, inability to walk, lack of coordination  7 = Loss of appetite  8 = Dullness | | 9 = Rough feathers  10 = Bluing of combs  11 = Sudden death  12 = Other *(Specify)* ****text box*** |
| **Newcastle disease** | | | |
| **J4** Have you ever had a Newcastle disease outbreak in your flock? | | | |
| 1 = Yes  2 = No | | | |
| **J5** How worried are you about Newcastle disease? | | | |
| 1 = Not at all worried  2 = A little worried  3 = Very worried | | | |
| **J6** How do you protect your chickens against Newcastle disease?  *(Do not read responses, select all that apply)* | | | |
| 1 = Vaccines  2 = Traditional medicine *(Specify)* ****text box***  3 = Human medicine eg: Paradone  4 = Keep chickens inside  5 = Slaughter if sick | | 6 = Sell if sick  7 = Clean coop  8 = Isolate new birds  9 = Other *(Specify)* | |
| **Vaccination** | | | |
| **J7** Are you aware that there is a vaccine for Newcastle disease? | | | |
| 1 = Yes  2 = No **(GO TO J27)** | | | |
| **J8** How did you first hear about the Newcastle disease vaccine?  *(Do not read responses, select best answer)* Allow one answer only | | | |
| 1 = Family member  2 = Friend  3 = Community member  4 = Livestock officer  5 = Chicken raising group  6 = Agrovet shop | | 7 = Seminar *(List the organizer)*  8 = Farmer field school  9 = NGO *(Specify)*  10 = Media *(Specify)*  11 = Agricultural exposition  12 = Other (*Specify)* | |
| **J9** Who is responsible for making decisions about whether or not to vaccinate the chickens? | | | |
| (Link to select person # from demographic chart) | | | |
| **J10** Have you ever vaccinated your chickens for Newcastle disease? | | | |
| 1 = Yes  2 = No **(GO TO J27)** | | | |
| **J11** Give the date of the last time you vaccinated. | | | |
| (mm/yyyy) | | | |
| **J12** What type of vaccine did you use the last time you vaccinated? | | | |
| 1 = Eye drop delivery (I-2)  2 = Drinking water delivery (La Sota)  3 = Other *(Specify)* | | | |
| **J13** *(Interviewer opinion)* Has the household vaccinated for Newcastle disease in the last **four months**? | | | |
| 1 = Yes  2 = No  3 = Not sure | | | |
| **J14** How old are the chickens when you first vaccinate them? | | | |
| 1 = Less than two weeks  2 = Two weeks to one month  3 = Older than one month | | | |
| **J15** Where do the vaccines you use come from? | | | |
| 1 = Agrovet shop  2 = Poultry group  3 = Livestock officer | | 4 = Funded organization (eg: World Vision)  5 = Other *(Specify)* | |
| **J16** Which of the following best describes how you get the Newcastle vaccine in your area? | | | |
| 1 = Community vaccinators come to my home to vaccinate my chickens.  2 = The vaccine is brought on an assigned day, and I administer myself.  3 = I buy the vaccine as needed and administer it myself.  4 = I don’t have a way to get vaccine.  5 = Other *(Specify)* | | | |
| **J17** How do you usually pay for the vaccine? | | | |
| 1 = Cash  2 = Contribution to group to purchase | | 3 = Vaccine is provided free **(GO TO J20)**  4 = Other (*Specify)* | |
| **J18** Did you share vaccine with anyone else the last time you vaccinates? | | | |
| 1 = Yes  2 = No | | | |
| **J19** How much in total did you pay to vaccinate your flock the last time you vaccinated? | | | |
| (Tsh) | | | |
| **J20** How many chickens did you vaccinate last time? | | | |
| (# of chickens) | | | |
| **J21** Did you vaccinate all of your chickens the last time you vaccinated? | | | |
| 1 = Yes  2 = No | | | |
| **J22** The last time you vaccinated, for how long did you travel to get the vaccine (one way)? | | | |
| 1 = Did not travel **(GO TO J25)**  2 = Less than one hour  3 = One to two hours  4 = More than two hours | | | |
| **J23** Did you transport the vaccine last time you bought it? | | | |
| 1 = Yes  2 = No **(GO TO J25)** | | | |
| **J24** Did you take any special measures to store while transporting it?  *(Read all responses, select all that apply)* | | | |
| 1 = Ice  2 = Wrapped with a wet cloth | | 3 = Thermos  4 = Other *(Specify)* | |
| **J25** Is the vaccine always available when you want it? | | | |
| 1 = Yes  2 = No  999 = Don’t know | | | |
| **J26** How do you track when the vaccine needs to be given?  *(Do not read responses, select all that apply)* | | | |
| 1 = Vaccinate when the vaccine is available in the village  2 = Vaccinate when others in the community vaccinate  3 = Member of household tracks in a notebook or on a calendar  4 = Member of household remembers when to vaccinate but does not formally write down  5 = When there is a disease outbreak  6 = When our chickens begin to show symptoms of disease  7 = Other *(Specify)* | | | |
| **J27** Do you vaccinate your chickens against any other diseases? | | | |
| 1 = Yes  2 = No **(GO TO J28)** | | | |
| **J27a** If yes, what diseases? | | | |
| 1 = Gumboro/ IBD  2 = Fowl pox  3 = Bronchitis  4 = Other *(Specify)* | | | |
| **J28** What are some factors that prevent you from vaccinating for Newcastle disease?  *(Do not read responses, select all that apply)* | | | |
| 1 = Price of vaccine  2 = Vaccines are far away  3 = Economic situation at home  4 = No expert  5 = Few chickens | | 6 = Fake vaccine  7 = Vaccine doesn’t work  8 = Little education  9 = Difficult to store vaccines  10 = Other *(Specify)* | |
| **J29** Do you think someone who vaccinates their flock will have a larger flock? | | | |
| 1 = Yes  2 = No  999 = Don’t know | | | |

| **SECTION K**: **KNOWLEDGE** |
| --- |
| *Answer the following questions.* |
| **K1** A vaccine can help a sick chicken to get better. |
| 1 = Yes  2 = No  999 = Don’t know |
| **K2** The Newcastle vaccine must be given once per year. |
| 1 = Yes  2 = No  999 = Don’t know |
| **K3** Newcastle disease has no treatment, it only has a vaccine. |
| 1 = Yes  2 = No  999 = Don’t know |
| **K4** The Newcastle vaccine protects chickens against all illnesses. |
| 1 = Yes  2 = No  999 = Don’t know |
| **K5** The Newcastle vaccine may not be effective if it isn’t stored properly. |
| 1 = Yes  2 = No  999 = Don’t know |

| **SECTION L: SOCIAL NETWORKS** | |
| --- | --- |
| **L1** Do you know anyone who vaccinates their chickens for Newcastle disease? | |
| 1 = Yes  2 = No | |
| **L2** What is your relationship with this person?  *(If knows multiple people, select the person the respondent knows best)* | |
| 1 = Family member  2 = Friend  3 = Neighbor | 4 = Community member  5 = Other *(specify)* |
| **L3** Is this person from this village? | |
| 1 = Yes  2 = No *(Specify location)* | |
| **L4** Who is the **main** person with whom you talk to about the health and care of your chickens?  *(Select one)* | |
| 1 = Family member  2 = Friend  3 = Neighbor  4 = Community member  5 = Livestock officer | 6 = Chicken raising group  7 = NGO representative  8 = Agrovet shop employee  9 = Other *(Specify)* |
| **L5** Is this person from this village? | |
| 1 = Yes  2 = No *(Specify location)* | |
| **L6** Is there a Livestock Officer in the area where you live? | |
| 1 = Yes  2 = No **(GO TO L9)** | |
| **L7** How often do you get information from the Livestock Officer about chicken raising? | |
| 1 = Often  2 = Occasionally  3 = Never | |
| **L8** How often do you get information from the Livestock Officer about livestock other than chickens? | |
| 1 = Often  2 = Occasionally  3 = Never | |
| **L9** Are there other chicken experts in the area where you live? | |
| 1 = Yes *(Specify)*  2 = No **(GO TO L11)** | |
| **L10** How often do you get information about chicken raising from other experts? | |
| 1 = Often  2 = Occasionally  3 = Never | |
| **L11** Have you ever gotten information about chicken raising from any of the sources listed below?  *(Read all sources listed and choose all that apply)* | |
| \| **Information source** \| 1 = Yes  2 = No \| \| --- \| --- \| \| Family member \|  \| \| Community member \|  \| \| Livestock raising group \|  \| \| NGO representative \|  \| \| Agrovet shop employee \|  \| \| Seminar *(List who hosted)* \|  \| \| Farmer field school \|  \| \| Agricultural exposition \|  \| \| Newspaper *(Which one?)* \|  \| \| Radio *(What station?)* \|  \| \| TV *(What channel / program)* \|  \| \| Internet *Specify)* \|  \| | |
| **L12** For each “Yes” response from L11 ask the following question  Is this information source present in the area where you live? | |
| 1 = Yes  2 = No *(List location)* | |
| **L13** For each “Yes” response from L11 ask the following question  How often do you get information from this source? | |
| 1 = Often  2 = Occasionally  3 = Never | |

| **SECTION M: WILLINGNESS TO PAY (WTP)** |
| --- |

*Note: Three variables will be randomized each day. The interviewer will enter them at the beginning of the section M of each survey, and question M1 will need to change accordingly. Questions M2-6 remain the same for all surveys.

Delivery system (1 OR 2)

Percent efficacy (70% OR 90%)

Change in bid (-1,500, -100, -500, +500, +1000, +1500) – See chart below. The initial bid is always 2,000/=.

***(Read introduction)*** This is the final section in the questionnaire. We will ask you what you are willing to pay for Newcastle disease vaccine. We are not selling or providing vaccine, we are conducting research to understand the need for vaccines in Tanzania. If you have any questions, please ask. It is fine to discuss with family members before answering.

**Delivery system 2:** The vaccine is available at the agrovet shop, and you need to go and purchase it yourself. The vaccine will protect your chickens against Newcastle disease for three months. The vaccine will be mixed into the chickens’ drinking water or delivered via eyedrops. Please remember the cost of the vaccine is in addition to other living costs that your household spends. **Suppose the vaccine has a ___% efficacy and protects each vaccinated chicken for three months.** For this example, assume you have ten chickens to vaccinate.

**Delivery System 1:** The vaccine service will be offered by trained community vaccinators who will come to your home. The vaccine will protect your chickens against Newcastle disease for three months. The vaccine will be mixed into the chickens’ drinking water or delivered via eyedrops. Please remember the cost of the vaccine is in addition to other living costs that your household spends. **Suppose the vaccine has a ___% efficacy and protects each vaccinated chicken for three months.** For this example, assume you have ten chickens to vaccinate.

| Did you understand the details of this vaccination program? Yes / No |
| --- |
| *If respondent answers NO to question 1, go back and explain - until you receive the answer YES.* Do not continue until yes is selected. This answer does not need to be recorded, it is a checkpoint to make sure the respondent understands the interviewer well. |
| **M1** If the cost to vaccinate TEN chickens is **___tsh**, are you willing to pay?  *Complete flow chart, and list the final amount household is willing to pay to vaccinate ten chickens.* |
| \| **Please see “Willingness to pay preferred format (section M)” attached as excel file for formatting** \| \| --- \| |

Willing to pay

2,000 tsh?

Willing to pay

___ tsh?

Willing to pay

____ tsh?

YES

NO

| **M2** At this price, would you choose to vaccinate all of the chickens in your flock? |
| --- |
| 1 = Yes, I would vaccinate all  2 = I would vaccinate some but not all  3 = No, I would not vaccinate any |
| **M3** Do you think it is reasonable for the manufacturer of the Newcastle disease vaccine to charge 60 tsh per chicken per dose? |
| 1 = Yes  2 = No |
| **M4** Would you be willing to pay part of the vaccine cost in advance of the day of vaccination so that the vaccine could be purchased? |
| 1 = Yes  2 = No  3 = Maybe |
| **M5** Which delivery system would you prefer? |
| 1 = Community vaccinators come to your home and vaccinate  2 = To buy and administer the vaccine yourself |
| **M6** What type of vaccine do you prefer? |
| 1 = Vaccine delivered via eyedrops  2 = Vaccine delivered via the chickens’ drinking water |

Thank you for your participation!
