# Supplementary material for: Why isn’t everyone using the thermotolerant vaccine? Preferences for Newcastle disease vaccines by chicken-owning households in Tanzania
Source: PLoS One. 2019 Aug 15;14(8):e0220963. doi: 10.1371/journal.pone.0220963 (PMC6695108; doi:10.1371/journal.pone.0220963)
Supplement: S2 Survey — (DOCX) [file pone.0220963.s002.docx]

*Note: This survey was programmed and administered electronically using Microsoft Visual Studio.*

**Programmer notes in green**

*Maelekezo kwa mtafiti*

**Maelekezo**

Tungependa kutambulisha mradi wetu wa utafiti kwenu na kujibu maswali yoyote mtakayokuwa nayo. Lengo kuu la mradi ni kuelewa mambo gani yanayoathiri wafugaji wa kuku wa kienyeji wanapofanya maamuzi yanayohusiana na chanjo ya maradhi ya mdondo. Tunataka wafugaji wa kuku waweze kupata huduma ambayo itawasaidia kuboresha ufugaji wa kuku wao.

Mradi unaofanywa kwa ushirikiano wa Taasisi ya Sayansi na Teknolojia ya Nelson Mandela jijini Arusha na Washington State University (Marekani). Mradi umefadhiliwa na Mfuko wa “Bill & Melinda Gates” uliopo nchini Marekani.

Tunahitaji mchango wako kwa sababu wewe ni mmojawapo wa wanajamii ambapo tunafanyia utafiti na maoni yako ni ya muhimu sana kwetu.

Ni matumaini yetu kwamba utashiriki kwenye mradi huu wa utafiti. Hata hivyo, uko huru kuamua kutoshiriki. Ukifanya maamuzi baada ya mradi kuanza kwamba hutaki tena kushirikishwa pia uko huru kujiondoa muda wowote.

Utafiti huu utatumia takribani dakika 45 ya muda wako. Mshiriki anapaswa awe na umri wa miaka 18 na kuendelea na awe na angalau kuku mmoja wa kienyeji AU awe amewahi kuwa na kuku miezi sita iliyopita.

Majibu yako yatakuwa siri. Na maelezo yako hayataripotiwa popote na majina. Je, utapenda kushiriki?

**Makubaliano na utambulisho wa mradi** (*Mtafiti: Nimemsomea mhusika mkuu utambulisho wa mradi na amekubali kuendelea.)*

**Click “yes” to continue**

| **SEHEMU A: MAELEZO YA UTANGULIZI** *(Ijazwe na mtafiti tu)* |
| --- |
| **A1** Siku, tarehe, mwezi na mwaka |
| (dd/mm/yyyy) |
| **A2** Jina la mtafiti |
|  |
| **A3** Mkoa:  Kijiji: Kitongoji: |
|  |

| **A4** Utambulisho wa kaya |
| --- |
|  |
| **A5** Je, kaya inastahili kuhusika katika mradi? |
| 1 = Ndio **(GO TO A7)**  2 = Hapana |
| **A6** Kama hapana, taja sababu. |
| 1 = Kaya haina kuku wa kienyeji sasa hivi wala haijawahi kuwa na kuku wa kienyeji miezi sita iliyopita  2 = Hakuna mwanakaya mtu mzima, angalau miaka 18  3 = kaya inafuga kuku wa kisasa tu  4 = Kukosa ushirikiano  5 = Nyinginezo *(Fafanua)* ****text box***  **(END SURVEY)** |
| **A7** Eneo la ‘GPS’ – (Latitude, longitude) |
| (Format: S ## **° ##.#### ’, E ### ° ##.#### ’** for example **S 03° 23.940’** **E 036 ° 47.958’)** |

| **SEHEMU B: SENSA YA KAYA** |
| --- |
| **B1** Orodhesha kila mwanakaya mwenye zaidi ya miaka 15.  *Jaza jedwali linalofuata.* |

|  | **Jinsia^1^** | **Umri**  **(miaka)** | **Hali ya ndoa^2^** | **Jukumu katika kaya^3^** | **Ngazi ya juu zaidi ya elimu^4^** |
| --- | --- | --- | --- | --- | --- |
| Mhusika wa kwanza (Anayejibu maswali) |  |  |  |  |  |
| Mhusika wa pili |  |  |  |  |  |
| Please allow additional spaces |  |  |  |  |  |
| ^1^**1** = Mwanaume, **2** = Mwanamke  ^2^**1** = Ameoa/Ameolea, **2 =** Hajaoa/Hajaolewa, **3 =** Mjane/Mgane, **4 =** Talaka  ^3^**1 =** Mkuu wa kaya; Uhusiano na mkuu wa kaya: **2** = mke/mume wake, **3** = mtoto wake, **4** = dada/kaka, **5** = mzazi, **6** = mfanyakazi, 7 = Nyinginezo *(Fafanua)*  ^4^**1** = Hajasoma, **2** = Elimu ya msingi, **3** = Elimu ya sekondari (kidato cha IV), **4** = Elimu ya sekondari (kidato cha VI), **5** = Elimu ya chuo, **6** = Elimu ya chuo kikuu | | | | | |

| **B2** Orodhesha watoto wa chini ya umri wa miaka 15 wanaoishi katika kaya.  *Jaza jedwali linalofuata.*  Response to B2 not required |
| --- |

| **Mhusika** | **Jinsia^1^** | **Umri** | **Anasoma shule? ^2^** |
| --- | --- | --- | --- |
| Mtoto wa kwanza |  |  |  |
| Mtoto wa pili |  |  |  |
| Please allow additional spaces |  |  |  |
| ^1^**1** = Kiume, **2** =Kike  **^2^1** = Ndiyo, **2** = Hapana | | | |

| **B3** Kabila la mkuu wa kaya? | | |
| --- | --- | --- |
| 1 = Mwarusha  2 = Mbena  3 = Mchaga  4 = Mgogo | 5 = Mhehe  6 = Iraqw  7 = Mmaasai  8 = Mnyiramba | 9 = Msangu  10 = Msukuma  11 = Lingine *(Fafanua)* |
| **B4** Kaya ina simu ya mkononi? | | |
| 1 = Ndiyo  2 = Hapana **(GO TO C1)** | | |
| **B5** Nambari ya simu: Response not required | | |
| Format: 9 digits | | |

| **SEHEMU C: RASILIMALI MIFUGO** |
| --- |
| **C1** Je, kaya hii inamiliki mifugo ya aina yeyote? |
| 1 = Ndiyo  2 = Hapana **(GO TO D1)** |
| **C2** Je, aina gani ya mifugo inamiliki kwa kaya? Wapo wangapi?  *Kwa aina ya mifugo isiyomilikiwa katika kaya, jaza 0.* |

Each space for “Total # of animals owned” required except “Other (Specify)”

| **Aina ya mfugo** | **Idadi ya wanyama (#)** |
| --- | --- |
| Ndama (chini ya miezi 12) |  |
| N’gombe dume |  |
| N’gombe jike |  |
| Mbuzi |  |
| Kondoo |  |
| Punda |  |
| Nguruwe |  |
| Bata |  |
| Kanga |  |
| Wengine *(Ainisha)* |  |

| **SEHEMU D: TAARIFA ZA KIPATO CHA KAYA** | | | | | | |
| --- | --- | --- | --- | --- | --- | --- |
| **D1:** Shughuli kuu ya kipato cha mkuu wa kaya ni ipi? | | | | | | |
| 1 = Kilimo  2 = Ufugaji  3 = Kibarua | | | | 4 = Biashara  5 = Ajira Serikalini au Shirika binafsi  6 = Nyinginezo *(fafanua)* | | |
| **D2:** Kuna mwanakaya yeyote aliyepata kipato kutoka katika shughuli zifuatazo **mwezi uliopita**?  *Soma majibu yote, chagua ndiyo au hapana.* | | | | | | |
| \|  \| **Aina ya shughuli** \| 1 = Yes  2 = No \| \| --- \| --- \| --- \| \| Kibarua \|  \| \| Ajira \|  \| \| Biashara \|  \| \| Malipo ya fedha yatokanayo na huduma mbalimbali \|  \| \| Malipo mengine kutoka kwenye miradi, serikalini, marafiki (ikiwemo mafao kama pensheni, misaada) \|  \| \| Mkopo kutoka benki au taasisi nyingine yoyote \|  \| \| Mkopo kutoka chanzo kisicho rasmi (mkopeshaji, ndugu) \|  \| \| Kupangisha eneo lako ikiwemo shamba lako au kupangisha majengo \|  \| \| Kukodisha wanyama wenu kwa shughuli za shamba \|  \| \| **Mauzo ya toka nayo na mazao ya kilimo** \| \| \| Mauzo ya mazao \|  \| \| Mauzo ya kuku \|  \| \| Mauzo ya mifugo wengine \|  \| \| Mauzo ya mayai \|  \| \| Mauzo ya bidhaa zingine kama maziwa, asali, ngozi \|  \| \| Nyinginezo (*Fafanua)* \|  \| | | | | | | |
|  | | | | | | |
| **D3:** Ni kipi kiasi kinachoonesha vizuri JUMLA ya kipato kilichopatikana katika kaya **mwezi uliopita**? Tafadhali, husisha vyanzo vyote vya kipato. | | | | | | |
| **1 =** 0 tsh  **2 =** 1-25,000 tsh  **3 =** 25,001-100,000 tsh  **4 =** 100,001-200,000 tsh  **5 =** 200,001-400,000 tsh  **6 =** 4000,001-700,000 tsh  **7 =** 700,001-1,000,000 tsh  **8 =** Zaidi ya 1,000,000 tsh  **777 =** Sina jibu | | | | | | |
| **D4:** Nyumba imejengwa kwa kutumia nini? *(Mtazamo wa mtafiti)* | | | | | | |
| 1 = Udongo au miti  2 = Matofali au sementi | | | | | | |
| **D5:** Sakafu imetengenezwa kwa kutumia nini? *(Mtazamo wa mtafiti)* | | | | | | |
| 1 = Udongo  2 = Cement | | | | 3 = Vigae  4 = Nyingenezo *(fafanua)* | | |
| **D6:** Paa imeezekwa kwa kutumia nini? *(Mtazamo ya mtafiti)* | | | | | | |
| 1 = Nyasi au makuti  2 = Bati  3 = Nailoni | | | | 4 = Vigae  5 = Nyinginezo *(Fafanua)* ****text box*** | | |
| **D7:** Je, nyumba inamilikiwa na kaya husika? | | | | | | |
| 1 = Ndiyo  2 = Hapana | | | | | | |
| **D8:** Je, nyumba inavyumba vingapi? | | | | | | |
| 1 = Chumba kimoja  2 = Vyumba viwili | | | | 3 = Vyumba vitatu  4 = Zaidi ya vyumba vitatu | | |
| **D9:** Je, ni aina gani ya jiko linalotumika kupikia? *(Chagua moja)* If 1 is selected, go to D 10. If not, skip to D 12 | | | | | | |
| 1 = Kuni  2 = Mkaa **(GO TO D12)**  3 = Mafuta taa **(GO TO D12)** | | 4 = Gesi **(GO TO D12)**  5 = Umeme **(GO TO D12)**  6 = Sola **(GO TO D12)** | | | 7 = Samadi ya ng’ombe **(GO TO D12)**  8 = Nyinginezo *(Fafanua)* **(GO TO D12)** | |
| **D10:** Kuni unazipata wapi? | | | | | | |
| 1 = Kununua **(GO TO D12)**  2 = Kukusanya | | | | | | |
| **D11:** Kwa ujumla, kaya inatumia muda gani kwa siku kukusanya kuni. | | | | | | |
| 1 = Chini ya saa moja  2 = Saa moja hadi masaa matatu  3 = Zaidi ya masaa matatu | | | | | | |
| **D12:** Mnatumia nini zaidi kwa ajili ya mwanga? *(Chagua moja)* | | | | | | |
| 1 = Umeme  2 = Mafuta ya taa  3 = Sola | | | | 4 = Taa ya betri  5 = Mshumaa  6 = Nyinginezo *(fafanua)* | | |
| **D13:** Je kaya inamiliki ardhi yoyote? | | | | | | |
| 1 = Ndiyo  2 = Hapana **(GO TO E1)** | | | | | | |
| **D14:** Ardhi ina ukubwa gani? | | | | | | |
| 1 = Chini ya hekari moja  2 = Hekari moja hadi tatu  3 = Zaidi ya hekari tatu | | | | | | |
|  | | | | | | |
| **SEHEMU E: MAJI NA USAFI** | | | | | | |
| **E1:** Je, kaya inatumia zaidi maji gani?  *(Chagua kila aina ya maji inayotumia)* | | | | | | |
| 1 = Maji ya bombani  2 = Mito na mifereji  3 = Maji ya mvua | | | 4 = Kisima  5 = Bwawa  6 = Chemchem | | | 7 = Nyinginezo *(Fafanua)* |
| **E2:** Je, unamiliki aina yoyote ya chanzo cha maji? (kama kisima) | | | | | | |
| 1 = Ndiyo  2 = Hapana | | | | | | |
| **E3:** Kaya inatumia masaa mangapi kuchota maji kwa ajili ya matumizi? | | | | | | |
| 1 = Chini ya saa moja  2 = Saa moja hadi masaa matatu  3 = Zaidi ya masaa matatu | | | | | | |
| **E4:** Kaya inatumia choo cha aina gani? | | | | | | |
| 1 = Shimo cha kizamani  2 = Shimo cha kisasa | 3 = Hakuna choo  4 = Choo cha kisasa cha ndani | | | | 6 = Nyinginezo *(Fafanua)* | |
| \| **SECTION F: USAFIRI** \| \| \| \| --- \| --- \| --- \| \| **F1** Wanakaya wanasafiri kwenda mjini au mji wowote wa karibu kama mara ngapi **kwa mwezi**? \| \| \| \| 1 = Hawendi  2 = Safari moja hadi tatu  3 = Safari nne au zaidi \| \| \| \| **F2** Njia gani ya usafiri inatumika zaidi kwenda mjini? (*Chagua moja)* \| \| \| \| 1 = Kutembea kwa miguu  2 = Baiskeli  3 = Pikipiki \| 4 = Daladala  5 = Bajaji  6 = Lori \| 7 = Gari  8 = Nyinginezo *(Fafanua)* \| | | | | | | |
| **SEHEMU G: CHAKULA** | | | | | | |
| **G1** Taja vyakula ambavyo vinazalishwa katika kaya. *(Chagua aina zote ambazo zinazalishwa)* | | | | | | |
| 1 = Nafaka (k.m. mahindi, mchele,mtama)  2 = Mboga mboga  3 = Nyama | | | | 4 = Maziwa  5 = Maharage, soya au kunde | | |
| **G2** Je, mnatumia nyama ya kuku nyumbani? | | | | | | |
| 1 = Ndiyo  2 = Hapana **(GO TO G5)** | | | | | | |
| **G3** Ni mara ngapi nyama ya kuku inaliwa nyumbani? | | | | | | |
| 1 = Kila wiki  2 = Kila mwezi | | | | 3 = Kila baada ya miezi sita  4 = Kila baada ya mwaka au zaidi | | |
| **G4** Nyama ya kuku inayotumika katika kaya inatoka wapi? | | | | | | |
| 1 = Kuku wanaofugwa nyumbani  2 = Inanunuliwa | | | | | | |
| **G5** Je, mnatumia mayai nyumbani? | | | | | | |
| 1 = Ndiyo  2 = Hapana **(GO TO G10)** | | | | | | |
| **G6** Ni mayai mangapi yaliliwa katika kaya **kwa kipindi cha siku tatu zilizopita**? | | | | | | |
| 1 = Hatujala **(GO TO G10)**  2 = Yai moja mpaka mayai matano | | | | 3 = Mayai sita mpaka kumi  4 = Zaidi ya mayai kumi | | |
| **G7** Mayai hayo yaliyoliwa yalizalishwa wapi? | | | | | | |
| 1 = Yote yalizalishwa nyumbani **(GO TO G10)**  2 = Yote yalinunuliwa | | | | 3 = Yalinunuliwa na kuzalishwa nyumbani | | |
| **G8** Ni mayai mangapi yalinunuliwa? | | | | | | |
| 1 = Yai moja mpaka matano  2 = Mayai sita mpaka kumi  3 = Zaidi ya mayai kumi | | | | | | |
| **G9** Ni wastani wa shilingi ngapi ililipwa kwa kila yai moja? | | | | | | |
| Sh ……… | | | | | | |

| **G10** Tafadhali zingatia milo iliyoliwa na watoto katika kaya kwa kipindi **cha masaa 72 yaliyopita (siku tatu)**. *Jaza 0 kama mtoto hajala mlo uliotanjwa hapo chini.* |
| --- |

Link to demographic chart for children in household so the additional three columns can be added to information already given regarding gender and age of children under 15 yrs.

| Mtoto | Idadi ya mayai ya kuku | Nyama ya kuku (Idadi ya milo) | Supu ya kuku (Idadi ya milo) |
| --- | --- | --- | --- |
|  |  |  |  |
|  |  |  |  |
|  |  |  |  |
|  |  |  |  |
|  |  |  |  |

| *Asome mtafiti:* Nitasoma maelezo kadhaa kwamba jinsi gani watu huandaa milo yao ya kila siku. Kwa maelezo yafuatayo, tafadhali niambie kama hali hiyo ilitokea mara kwa mara, ilitokea mara chache, haikutokea kabisa (wewe au kaya yako) kwa miezi kumi na mbili iliyopita, kwamba tangu (jina la mwezi husika). |
| --- |
| **G11** Maelezo ya kwanza ni “Chakula kilichozaalishwa au kununuliwa hakikutosheleza, na hatukuwa na njia kupata kingine zaidi. Tafadhali niambie kama hali hiyo ilitokea mara kwa mara, ilitokea mara chache, haikutokea kabisa (wewe au kaya yako) kwa miezi kumi na mbili iliyopita. |
| 1 = Ilitokea mara kwa mara  2 = Ilitokea mara chache  3 = Haikutokea  999 = Hajui  777 = Amekataa kujibu |
| **G12** “Hatukuweza kumudu kula aina mbalimbali za vyakula katika milo?” Tafadhali niambie kama hali hiyo ilitokea mara kwa mara, ilitokea mara chache, haikutokea kabisa (wewe au kaya yako) kwa miezi kumi na mbili iliyopita. |
| 1 = Ilitokea mara kwa mara  2 = Ilitokea mara chache  3 = Haikutokea  999 = Hajui  777 = Amekataa kujibu |
| **G13** Katika miezi kumi na mbili iliyopita, tangu (jina la mwezu husika), je, wewe au watu wazima wengine katika kaya yako, walipunguza chakula au kuruka mlo kwa sababu hakukuwa na chakula au pesa kwa ajili ya kununua chakula. |
| 1 = Ndiyo  2 = Hapana **(GO TO G15)**  3 = Hajui **(GO TO G16)** |
| **G14** Ni mara ngapi ilitokea? |
| 1 = Karibu kila mwezi  2 = Baadhi ya miezi lakini si kila mwezi  3 = Mwezi moja au miezi miwili tu  999 = Hajui |
| **G15** Katika miezi kumi na mbili iliyopita, je, umewahi kula kiasi kidogo cha chakula ambacho unadhani hukutakiwa kula kwasababu hakuna chakula cha kutosha au pesa za kutosha kwa ajili ya chakula. |
| 1 = Ndiyo  2 = Hapana  999 = Hajui |
| **G16** Katika miezi kumi na mbili iliyopita, je, umewahi kusikia njaa lakini hukula kwa sababu hakukuwa na chakula cha kutosha au pesa za kutosha kwa ajili ya chakula. |
| 1 = Ndiyo  2 = Hapana  999 = Hajui |
| **SEHEMU H: GHARAMA ZA ELIMU NA MATIBABU** |
| **H1** Ni shilingi ngapi zimetumika kwa kipindi cha **miezi minne** iliyopita kwa ajili ya ada za wanafunzi? |
| Sh. |
| **H2** Ni shilingi ngapi zimetumika kwa kipindi cha **miezi minne** iliyopita kwa ajili ya matibabu na gharama nyingine kama kwenda kliniki, kulazwa, dawa au usafiri wa kwenda kituo cha afya? |
| Sh. |

| **SEHEMU I: KUKU** |
| --- |
| **I1** Nani ni muhusika mkuu katika kutoa maamuzi kuhusu kuku?  *(Kama hapana, uombe kuongea na mhusika mkuu kama anapatikana)* |
| (Allow choice of people from demographic chart, multiple selection possible) |
| **I1a** Huyu mtu anapatikana ili kuendelea na mahojiano? |
| 1 = Ndiyo  2 = Hapana |
| **I2** Vifaranga, matetea au jogoo wangapi wanamilikiwa na kaya?  *Jaza jedwali chini. Kwa vifaranga (wenye chini ya miezi mitatu) kadiria kwa tano.* |

|  | **Idadi (#)** |
| --- | --- |
| Jogoo |  |
| Matetea |  |
| Vifaranga (chini ya miezi mitatu) |  |

| **I3** Katika banda lako, unafuga kuku wa aina gani?  *(Chagua majibu yote yanayofaa)* |
| --- |
| 1 = Kuku wa kienyeji  2 = Kuku wa kisasa wa mayai  3 = Kuku wa kisasa wa nyama |
| **I4** Maswali yafuatayo yanahusu kupungua na kuongezeka kwa kuku wako katika kaya yako.  *Tafadhali jaza jedwali kuhusu uingizwaji wa kuku katika kaya kwa kipindi cha* ***mwezi uliopita****. Orodhesha namba ya kila mmoja. Kwa vifaranga, kadiria kwa tano.*  Each non-shaded box requires an answer, prompt to write zero if not applicable. |

|  | Jogoo | Matetea | Vifaranga (chini ya miezi mitatu) | **JUMLA** |
| --- | --- | --- | --- | --- |
| Waliototolewa |  |  |  |  |
| Walionunuliwa |  |  |  |  |
| Walioletwa kama zawadi |  |  |  |  |
| **Jumla ya idadi iliyoongezeka** |  |  |  |  |
|  | | | | |
| Waliouzwa |  |  |  |  |
| Waliotolewa kama zawadi |  |  |  |  |
| Walioshambuliwa na wanyama |  |  |  |  |
| Walioibwa |  |  |  |  |
| Waliokufa na kuliwa |  |  |  |  |
| Waliokufa na kutupwa |  |  |  |  |
| Waliochinjwa na kuliwa |  |  |  |  |
| **Jumla ya idadi iliopungua** |  |  |  |  |

| **I5** Kwa nini mnafuga kuku? *(Chagua majibu yanayofaa)* | | | |
| --- | --- | --- | --- |
| 1 = Kipato kutokana na kuuza kuku  2 = Kipato kutokana na kuuza mayai  3 = Matumizi madogo madogo nyumbani  4 = Kutatua matatizo | | 5 = Mboga  6 = Wageni  6 = Mengineyo *(Fafanua)* | |
| **I6** Chagua njia ambayo unatumia kufuga kuku wako. | | | |
| 1 = Wanazunguka nje hadi jioni  2 = Wanazunguka nje na kuingia ndani jioni | | 3 = Wanafungiwa ndani wakati wote na wanapewa chakula | |
| **I7** Kuku wanalishwa chakula kilichoongezewa virutubisho? (kama mifupa, madini, mashudu, mabaki ya dagaa) | | | |
| 1 = Kila siku  2 = Kila wiki | | 3 = Mara moja au mbili kwa mwezi  4 = Hawalishwi | |
| **I8** Umekuwa ukifuga kuku kwa muda gani? | | | |
| 1 = Chini ya mwaka mmoja  2 = Mwaka mmoja mpaka miaka mitano | | 3 = Zaidi ya miaka mitano | |
| **I9** Ni nani anayehusika na shughuli za kila siku za kutunza kuku? *(Chagua mtu mmoja)* | | | |
| 1 = Mtu ambaye anaishi hapa (Link to drop down menu with info from demographic chart to enumerator can choose appropriate person # )  2 = Mfanyakazi | | | |
| **I10** Ni nani anayehusika kuamua kuku anunuliwe au auzwe? *(Chagua wote wanaofaa)* | | | |
| (Link to drop down menu with info from demographic chart so interviewer can choose appropriate person #. Allow selection of two people maximum. ) | | | |
| **I11** Ni wapi unauzia mayai? *(Chagua majibu yote yanayofaa)* | | | |
| 1 = Dukani  2 = Sokoni  3 = Vibanda vya chipsi  4 = Mgahawani/ Hotelini | 5 = Barabarani  6 = Wachuuzi  7 = Kutembeza mitaani  8 = Watu binafsi | | 9 = Stesheni  10 = Kanisani/ Msikitini  11 = Sherehe  12 = Hatuuzi mayai  13 = Mengineyo *(Fafanua)* |
| **I12** Ni wapi unauzia kuku? *(Chagua majibu yote yanayofaa)* | | | |
| 1 = Dukani  2 = Sokoni  3 = Hotelini au migahawani | | 4 = Wateja wanakuja nyumbani kununu  5 = Hatuuzi mayai  6 = Nyingenezo (*Fafanua)* | |
| **I13** Ni kuku wangapi ulionao wanataga mayai? | | | |
| (Idadi ya kuku) | | | |
| **I14** Kuku wanataga wastani wa mayai mangapi kwa wiki? | | | |
| (Idadi ya mayai) | | | |
| **I15** Ni mayai mangapi unauza kwa wiki? **If 0, skip to I17** | | | |
| (Idadi ya mayai)--- | | | |

| **I16** Je unapata wastani wa shilingi ngapi kwa kuuza yai moja la kuku wa kienyeji? | | |
| --- | --- | --- |
| Sh--- | | |
| **I17** Unauza kuku wa kienyeji wangapi kwa mwezi? **If 0, skip to I 19** | | |
| (Idadi ya kuku) | | |
| **I18** Unapata wastani wa shilingi ngapi kwa kuuza kuku mmoja wa kienyeji? | | |
| Sh--- | | |
| **I19** Ni sababu zipi zinakufanya wewe kutoongeza idadi kubwa ya kuku?  *(Baada ya kumuliza sikiliza majibu yake na oanisha na majibu ya kwenye orodha. Chagua yote yanayofaa.)* | | |
| 1 = Magonjwa  2 = Kuuza kuku  3 = Wizi  4 = Kushambuliwa na wanyama  5 = Vifaa kwa vifaranga | 6 = Eneo la kufuga halitoshi  7 = Ufugaji wa kienyeji  8 = Mayai machache / kuku hawatagi vizuri  9 = Kutotumia chanjo | 10 = Kutofuata ratiba ya chanjo  11 = Elimu ndogo  12 = Uzembe  13 = Nyingenezo *(Fafanua)* |
| **I20** Vifaranga wanapatikana katika maeneo mnayoishi? | | |
| 1 = Ndiyo  2 = Hapana  999 = Hajui | | |
| **I21** Je, kufuga kuku ni biashara yenye faida katika maeneo mnayoishi? | | |
| 1 = Ndiyo  2 = Hapana | | |
| **I22** Je, wewe ni mmoja wapo wa kikundi cha wafugaji wa kuku? | | |
| 1 = Ndiyo  2 = Hapana | | |

| **SEHEMU J: HUDUMA YA CHANJO NA MARADHI YA MDONDO/ KIDERI** | | | | |
| --- | --- | --- | --- | --- |
| **Historia:** | | | | |
| **J1** Katika kipindi cha **miezi sita** iliyopita, je, theluthi au zaidi ya kuku walikufa? | | | | |
| 1 = Ndiyo  2 = Hapana **(GO TO J4)** | | | | |
| **J2** Unafikiri nini kilisababisha vifo hivyo?  *(Baada ya kumuliza, sikiliza majibu yake na oanisha na majibu ya kwenye orodha. Chagua moja.)* | | | | |
| 1 = Kideri / mdondo  2 = Ndui (Fowl Pox)  3 = Gumboro/ Infectious Bursal Disease | | 4 = Nyingenezo *(Fafanua)*  999 = Hajui | | |
| **J3** Unaweza kutambua dalili yoyote kati ya zifuatazo wakati wa vifo hivyo?  *(Soma machagulio yote, chagua yote yanayofaa.)* | | | | |
| 1 = Kukohoa  2 = Chafya  3 = Kupumua kwa shida  4 = Kutoa kamasi puani | 5 = Kuhara  6 = Matatizo ya neva za fahamu k.m kunyonga shingo, kunyongea, kutotembea, kupepesuka  7 = Kukosa hamu ya kula  8 = Kutochangamka | | | 9 = Kuharibika kwa manyoya  10 = Kilemba (comb) kubadilika rangi ya bluu  11 = Vifo vingi vya ghafla  12 = Nyingenezo *(Fafanua)* |
| **Mdondo/ Kideri** | | | | |
| **J4** Je, umewahi kuwa na kuku walioumwa mdondo/ kideri? | | | | |
| 1 = Ndiyo  2 = Hapana | | | | |
| **J5** Je, una mashaka kiasi gani kuhusu maradhi ya mdondo/kideri? | | | | |
| 1 = Sina mashaka  2 = Nina mashaka kidogo  3 = Nina mashaka sana | | | | |
| **J6** Je, unafanya nini kuwakinga kuku wako na maradhi ya mdondo/kideri?  *(Baada ya kumuliza, sikiliza majibu yake na oanisha na majibu ya kwenye orodha. Chagua yote yanayofaa.)* | | | | |
| 1 = Chanjo  2 = Tiba ya asili *(Fafanua)*  3 = Dawa za wanadamu k.m. Paradone  4 = Kuwafungia ndani  5 = Kuchinja kuku wanaoumwa | | 6 = Kuuza kuku wanoumwa  7 = Usafi wa banda  8 = Kutenga kuku wapya pekee yao  9 = Nyingenezo *(Fafanua)* | | |
| **Matumizi ya Chanjo** | | | | |
| **J7** Je, unajua kama kuna chanjo ya kisasa kuzuia ugonjwa wa mdondo/ kideri? | | | | |
| 1 = Ndiyo  2 = Hapana **(GO TO J27)** | | | | |
| **J8** Ulisikia kutoka wapi kwa mara ya kwanza kuhusu chanjo ya mdondo/kideri?  *(Usimsomea majibu, sikiliza jibu lake na oanisha na jibu la kwenye orodha. Chagua jibu moja linalofaa.)* | | | | |
| 1 = Mwanafamilia  2 = Rafiki  3 = Mwanajamii  4 = Afisa mifugo  5 = Kikundi cha wafugaji wa kuku  6 = Duka la dawa za mifugo | | | 7 = Semina *(Iliyotolewa na nani? Fafanua)*  8 = Shamba darasa  9 = Shirika *(Fafanua)*  10 = Vyombo vya habari *(Fafanua)*  11 = Nane Nane  12 = Nyingenezo (*Fafanua)* | |
| **J9** Nani anawajibika kufanya maamuzi ya kuchanja au kutokuchanja kuku? | | | | |
| (Link to select person # from demographic chart) | | | | |
| **J10** Je, umewahi kuwapa kuku wenu chanjo ya kideri? | | | | |
| 1 = Ndiyo  2 = Hapana **(GO TO J27)** | | | | |
| **J11** Taja tarehe ya mwisho uliyochanja kuku. | | | | |
| (mm/yyyy) | | | | |
| **J12** Ni aina gani ya chanjo uliyotumia mara ya mwisho kuchanja kuku wenu? | | | | |
| 1 = Kuweka matone machoni (I-2)  2 = Ya kuchanganya kwenye maji ya kunywa (La Sota)  3 = Nyingenezo *(Fafanua)* | | | | |
| **J13** *(Ijazwe na mtafiti)* Ndani ya **miezi minne** iliyopita, kuku wa kaya wamepata chanjo ya kideri? | | | | |
| 1 = Ndiyo  2 = Hapana  3 = Haieleweki | | | | |
| **J14** Chanjo ya kwanza inatolewa kwa kuku wenye umri gani? | | | | |
| 1 = Chini yawiki mbili  2 = Wiki mbili hadi mwezi moja  3 = Zaidi ya mwezi moja | | | | |
| **J15** Chanjo unayotumia inatoka wapi? | | | | |
| 1 = Duka la dawa za mifugo  2 = Kikundi cha wafugaji wa kuku  3 = Afisa Mifugo | | | 4 = Shirika (k.m. World Vision)  5 = Nyingezo *(Fafanua)* | |
| **J16** Ni ipi kati ya njia zifuatazo inaelezea vizuri namna mnavyopata chanjo ya mdondo kwenye maeneo mnayoishi? | | | | |
| 1 = Watoa chanjo waliosomea huja nyumbani kwangu mara tatu kwa mwaka kutoa chanjo hii.  2 = Chanjo huletwa kijijini siku maalum, kisha nawapa kuku chanjo mwenyewe nyumbani.  3 = Nanunua chanjo ninapotaka na kuwapa kuku wangu mwenyewe.  4 = Hajui namna ya kupata chanjo.  5 = Njia nyingine *(Fafanua)* | | | | |
| **J17** Je, unalipiaje chanjo? | | | | |
| 1 = Fedha taslim  2 = Mchango wa kikundi kwa ajili ya manunuzi ya chanjo | | | 3 = Inatolewa bure **(GO TO J20)**  4 = Nyinginezo *(Fafanua)* | |
| **J18** Je, mara ya mwisho kununua chanjo uligawa chanjo na mtu mwingine yeyote? | | | | |
| 1 = Ndiyo  2 = Hapana | | | | |
| **J19** Je, mara ya mwisho kutoa chanjo, ulilipa shilling ngapi kwa ujumla? | | | | |
| (Sh) | | | | |
| **J20** Je, mara ya mwisho kutoa chanjo, ulichanja kuku wangapi? | | | | |
| (Idadi ya kuku) | | | | |
| **J21** Je, mara ya mwisho kutoa chanjo, ulichanja kuku wote? | | | | |
| 1 = Ndiyo  2 = Hapana | | | | |
| **J22** Je, ulipokwenda kufuata chanjo mara ya mwisho, ulitumia muda gani kusafiri (kwenda tu)? | | | | |
| 1 = Hakusafari **(GO TO J25)**  2 = Chini ya saa moja  3 = Saa moja hadi masaa mawili  4 = Zaidi ya masaa mawili | | | | |
| **J23** Ulisafirisha chanjo mara ya mwisho ulipochanja? | | | | |
| 1 = Ndiyo  2 = Hapana **(GO TO J25)** | | | | |
| **J24** Je, ulichukua tahadhari yoyote ya uhifadhi wakati wa usafirishaji?  *(Soma machagulio yote, chagua yote yanayofaa)* | | | | |
| 1 = Barafu  2 = Njia ya kulowanisha kipande cha nguo | | 3 = Chupa ya chai  4 = Nyingenezo *(Fafanua)* | | |
| **J25** Chanjo hupatikana wakati wowote unapoihitaji? | | | | |
| 1 = Ndiyo  2 = Hapana  999 = Hajui | | | | |
| **J26** Je, ni muda gani hutoa chanjo kwa kuku wako?  *(Baada ya kumuliza, sikiliza majibu yake na oanisha na majibu ya kwenye orodha. Chagua zote zinazotumika)* | | | | |
| 1 = Tunatoa chanjo wakati chanjo inapatikana kijijini  2 = Tunatoa chanjo wakati wengine wanapotoa chanjo katika jamii  3 = Mwanakaya anarekodi katika daftari au kalenda  4 = Kuna mwanakaya anakumbuka wakati wa kutoa chanjo ila haiaandikwi rasmi  5 = Wakati mlipuko wa ugonjwa unapotokea  6 = Wakati kuku wenu wanapoonyesha dalili za ugonjwa  7 = Nyinginezo *(Fafanua)* | | | | |
| **J27** Je, unachanja kuku wako dhidi ya magonjwa mengine? | | | | |
| 1 = Ndiyo  2 = Hapana **(GO TO J28)** | | | | |
| **J27a** Kama ndiyo, ni magonjwa yapi? | | | | |
| 1 = Gumboro/ IBD  2 = Ndui (Fowl Pox)  3 = Mafua (Bronchitis)  4 = Nyingenezo *(Fafanua)* | | | | |
| **J28** Ni sababu gani zinazowazuia kuchanja kuku wenu?  *(Usimsomea majibu, sikiliza majibu yake na oanisha majibu yake kwenye orodha. Chagua yote yanyofaa.)* | | | | |
| 1 = Bei ya chanjo  2 = Ubali wa huduma  3 = Hali ya uchumi ya familia  4 = Hakuna mtalaamu  5 = Kuku ni wachache | | 6 = Chanjo feki  7 = Chanjo hazifanyi kazi  8 = Elimu ndogo  9 = Ngumu kuhifadhi chanjo  10 = Nyingenezo *(Fafanua)* | | |
| **J29** Je, unadhani anayechanja kuku wake atakuwa na kuku wengi zaidi? | | | | |
| 1 = Ndiyo  2 = Hapana  999= Hajui | | | | |

| **SEHEMU K: UJUZI** |
| --- |
| *Jibu maswali yanyofuata.* |
| **K1** Chanjo inaweza kusaidia kuku anayeumwa kupata nafuu. |
| 1 = Ndiyo  2 = Hapana  999 = Hajui |
| **K2** Chanjo dhidi ya ugonjwa wa kideri lazima itolewe mara moja kwa mwaka. |
| 1 = Ndiyo  2 = Hapana  999 = Hajui |
| **K3** Ugonjwa wa kideri hauna tibu, ila kinga tu. |
| 1 = Ndiyo  2 = Hapana  999 = Hajui |
| **K4** Chanjo dhidi ya ugonjwa wa kideri kuwa kinga kuku dhidi ya magonjwa yote. |
| 1 = Ndiyo  2 = Hapana  999 = Hajui |
| **K5** Chanjo dhidi ya mdondo inaweza isifanye kazi endapo haitahifadhiwa kwa usahihi. |
| 1 = Ndiyo  2 = Hapana  999 = Hajui |

| **SEHEMU L: TAARIFA ZA KIJAMII** | |
| --- | --- |
| **L1** Je, unamjua yeyote anayechanja kuku wake dhidi ya mdondo/kideri? | |
| 1 = Ndiyo  2 = Hapana | |
| **L2** Una uhusiano gani na huyu mtu?  *(Kama anawafahamu watu zaidi, mchagulie mtu mmoja anyejua vizuri zaidi.)* | |
| 1 = Mwanafamilia  2 = Rafiki  3 = Jirani | 4 = Mwanajamii  5 = Mwingine *(Fafanua)* |
| **L3** Mtu huyu anaishi kijijini hapa? | |
| 1 = Ndiyo  2 = Hapana (*Taja eneo)* | |
| **L4** Unazungumza na nani **zaidi** kuhusu afya ya kuku wako?  *(Chagua moja)* | |
| 1 = Mwanafamilia  2 = Rafiki  3 = Jirani  4 = Mwanajamii  5 = Afisa mifugo | 6 = Kikundi cha wafugaji  7 = Mtoa huduma wa shirika zisizo za kiserikali  8 = Mfanyakazi wa duka la dawa za mifugo  9 = Mwingine (*Fafanua)* |
| **L5** Mtu huyu anaishi kijijini hapa? | |
| 1 = Ndiyo  2 = Hapana *(Taja eneo)* | |

| **L6** Je, kuna Afisa Mifugo katika maeneo mnayoishi? |
| --- |
| 1 = Ndiyo  2 = Hapana **(GO TO L9)** |
| **L7** Hupata taarifa kutoka kwa Afisa Mifugo kuhusu ufugaji wa kuku? |
| 1 = Mara kwa mara  2 = Mara chache  3 = Sijawahi kupata taarifa |
| **L8** Hupata taarifa kutoka kwa Afisa Mifugo kuhusu ufugaji wa mifugo wengine? |
| 1 = Mara kwa mara  2 = Mara chache  3 = Sijawahi kupata taarifa |
| **L9** Kuna wataalum wengine wa kuku katika maeneo mnayoishi? |
| 1 = Ndiyo *Fafanua.*  2 = Hapana **(GO TO L11)** |
| **L10** Je, unapata taarifa kuhusu ufugaji wa kuku kutoka kwa wataalum wengine? |
| 1 = Mara kwa mara  2 = Mara chache  3 = Sijawahi kupata taarifa |
| **L11** Umewahi kupata taarifa kuhusu ufugaji wa kuku kutoka katika orodha ifuatayo?  *(Soma majibu yote na chagua yote yanayofaa)* |
| \| **Chanzo ya taarifa** \| 1 = Ndiyo  2 = Hapana \| \| --- \| --- \| \| Mwanafamilia \|  \| \| Mwanajamii \|  \| \| Kikundi cha wafugaji \|  \| \| Mtoa huduma wa shirika zisizo za kiserikali \|  \| \| Mfanyakazi wa duka la dawa za mifugo \|  \| \| Seminar *(Imetolewa na nani?)* ****text box*** \|  \| \| Shamba darasa \|  \| \| Nane Nane \|  \| \| Gazeti *(Ipi?)* ****text box*** \|  \| \| Redio *(Stesheni gani?)* ****text box*** \|  \| \| TV *(Fafanua)* ****text box*** \|  \| \| Mtandoa *(Fafanua)* ****text box*** \|  \| |
| **L12** For each “Yes” response from L11 ask the following question  Chanzo hiki cha taarifa kipo maeneo mnayoishi? |
| 1 = Ndiyo  2 = Hapana *(Taja eneo)* ****text box*** |
| For each “Yes” response from L11 ask the following question  **L13** Je, unapata taarifa kutoka katika chanzo hiki? |
| 1 = Mara kwa mara  2 = Mara chache  3 = Hajawahi kupata taarifa |

| **SEHEMU M: UKUBALI WA MALIPO** |
| --- |

*Note: Three variables will be randomized each day. The interviewer will enter them at the beginning of the section M of each survey, and question M1 will need to change accordingly. Questions M2-6 remain the same for all surveys.

Delivery system (1 OR 2)

Percent efficacy (70% OR 90%)

Change in bid (-1,500, -100, -500, +500, +100, +1500). The initial bid is always 2,000/=.

***(Asome mtafiti)*** Hiki ni kifungu cha mwisho katika maswali. Tutakuuliza kuhusu utayari wako wa kulipa kwa ajili ya chanjo za kideri. Hatutengenezi wala hatuuzi chanjo, tufanya utafiti huu ili tuelewe mahitaji ya chanjo hapa Tanzania. Kama una swali lolote, tafadhali uliza. Inaruhusiwa kufanya majadiliano na familia ili kupata uelewe.

**Njia ya kwanza ya utoaji:** Huduma ya chanjo itatolewa na wanajamii waliofundishwa. Watakuja nyumbani kwako mara tatu kwa mwaka na watachanja kuku wote. Kuchanja mara tatu kwa mwaka ni muhimu kwa kuzuia ugonjwa. Chanjo hii itawapa kinga kuku wako dhidi ya maradhi ya mdondo/kideri. Chanjo itakoyotolewa ni ya kunywesha kwenye maji au matone kwenye jicho.

Huduma hii itakuwa ya malipo kwa kaya ambapo itatakiwa kulipa ili upate faida. Tafadhali kumbuka hii ni nyongeza ya gharama zingine sa maisha ambayo kaya inagharimia. **Tuseme chanjo ina uwezo wa kukinga kuku kwa asilimia____na inafanya kazi kwa kipindi cha miezi mitatu.** Kwa maneno mengine, ugonjwa ukija, asilimia __ ya kuku watakingwa na hawataumwa mdondo / kideri. Kwa ajili ya mafano huu, tuseme una kuku kumi nyumbani.

**Njia ya pili ya utoaji:** Chanjo inapatikana kwenye duka la dawa za mifugo, na ina bidhii uende kununua mwenyewe. Kuchanja mara tatu kwa mwaka ni muhimu kwa kuzuia ugonjwa. Chanjo hii itawapa kinga kuku wako dhidi ya maradhi ya mdondo/kideri. Chanjo itakoyotolewa ni ya kunywesha kwenye maji au matone kwenye jicho.

Huduma hii itakuwa ya malipo kwa kaya ambapo itatakiwa kulipa ili upate faida. Tafadhali kumbuka hii ni nyongeza ya gharama zingine sa maisha ambayo kaya inagharimia. **Tuseme chanjo ina uwezo wa kukinga kuku kwa asilimia____na inafanya kazi kwa kipindi cha miezi mitatu.** Kwa maneno mengine, ugonjwa ukija, asilimia __ ya kuku watakingwa na hawataumwa mdondo / kideri. Kwa ajili ya mafano huu, tuseme una kuku kumi nyumbani.

| Je, umeelewa maelezo ya huduma ya chanjo? |
| --- |
| *Kama jibu ni hapana, rudi na eleza mpaka jibu liwe ndiyo.* Do not continue until yes is selected. This answer does not need to be recorded, it is a checkpoint to make sure the respondent understands the interviewer well. |
| **M1** Je, kama gharama ya chanjo ni **sh____**kwa kuku **KUMI**, uko tayari kulipa?  *Jaza jedwali na orodhesha kiasi cha mwisho cha pesa ambacho kaya inaweza kulipa kwa kuchanja kuku kumi*. |
|  |

Uko tayari kulipa Sh

2000?

**Uko tayari kulipa Sh**

**___?**

**Uko tayari kulipa Sh**

**___?**

NDIYO

HAPANA

| **M2** Kwa bei hii, ungeweza kuchagua kuwapa chanjo kuku wote kwenye banda lako? |
| --- |
| 1 = Ningechanja kuku wote  2 = Ningechanja wachache lakini siyo wote  3 = Nisingechanja kuku yeyote |
| **M3** Unafikiri ni sawa kwa mtengenezaji wa chanjo ya maradhi ya mdondo/kideri kulipisha sh 60 kwa kuku kwa dozi moja? |
| 1 = Ndiyo  2 = Hapana |
| **M4** Unaweza kuwa tayari kulipa sehemu ya gharama ya chanjo mapema kabla ya siku husika ili chanjo iweze kununuliwa? |
| 1 = Ndiyo  2 = Hapana  3 = Labda |
| **M5** Unapendelea utaratibu gani wa kutoa huduma ya chanjo? |
| 1 = Kutoa chanjo kwa kutumia waatalam ngazi ya jamii  2 = Kujisimamia wewe mwenyewe kwa kununua chanjo na kuchanja kuku wako mwenyewe |
| **M6** Je, ungependelea aina gani ya chanjo? |
| 1 = Chanjo ya matone  2 = Chanjo ya kuchanganya kwenye maji ya kunywa kuku |

Asante kwa ushirikiono!
