# Supplementary material for: Why isn’t everyone using the thermotolerant vaccine? Preferences for Newcastle disease vaccines by chicken-owning households in Tanzania
Source: PLoS One. 2019 Aug 15;14(8):e0220963. doi: 10.1371/journal.pone.0220963 (PMC6695108; doi:10.1371/journal.pone.0220963)
Supplement: S1 Text — (DOCX) [file pone.0220963.s003.docx]

S1 Text. Focus group questions.

*One facilitator plus two note-keepers.*

Village: _____________________________________________________

Date (dd/mm/yyyy): __________________________________________

Enumerator name: ___________________________________________

Focus group demographics: *(Write or tally number of participants)*

| Female | Men |
| --- | --- |
|  |  |

| Youth (18-35 yrs) | Middle age (36-55 yrs) | Elders (55+ yrs) |
| --- | --- | --- |
|  |  |  |

Guests in attendance (e.g.: Livestock Officer, Village Executive Officer)

______________________________________________________________________________

*Instructions: Remind research assistants and participants that there are no correct answers. Can also answer with beliefs or opinions within the community even if it is not something they personally believe or do. Our goal is to understand how people view vaccines and how they choose between two types of vaccines.*

Disease and vaccines:

List some things that you think can cause people to get sick.

List some things that you think can cause livestock to get sick.

What kinds of things can people do to avoid getting sick?

What kinds of things can people do to keep livestock from getting sick?

What do you think disease is made of?

What do you think a vaccine is (in your opinion)?

How do you think a vaccine works?

What do you think a vaccine is made of?

Vaccines work for illnesses such as______.

What qualities does a good vaccine have?

What are some ways vaccines can be administered? *(Fill the table below)*

What are pros and cons to each administration style listed? *(Fill below)*

| Administration style | Pro | Con |
| --- | --- | --- |
| 1. Drinking water |  |  |
| 1. Eye drop |  |  |
| 3. |  |  |

ND vaccines:

What types of Newcastle disease (ND) vaccines are in Tanzania?

*If not known by group, clarify that there are two types of ND vaccine we will discuss going forward: I-2 (eye drop) and La Sota (drinking water).*

What do you consider when deciding which vaccine to use?

Has anyone used both types? How was it? Why did you switch?

For those who have only used one type, would you be willing to switch? Why or why not?

How important are the following items to you when considering whether or not to use a vaccine?

*(No need to create consensus; document the conversations only.)*

1. Price
2. Distance to travel to buy
3. Availability
4. Know someone who vaccinates
5. Ease of sharing
6. Fast to use
7. Easy to use
8. Administration type
9. Thermotolerance
10. Made in Tanzania or imported

Comparing La Sota and I-2

Which is easier to share?

Which is more effective and why?

Which is less expensive in this area? Has that always been true?

Which is easier to use? Why?

Which is faster to use? Why?

Which is better for a farmer with fewer chickens? Why?

Additional information?
