# Supplementary material for: Why isn’t everyone using the thermotolerant vaccine? Preferences for Newcastle disease vaccines by chicken-owning households in Tanzania
Source: PLoS One. 2019 Aug 15;14(8):e0220963. doi: 10.1371/journal.pone.0220963 (PMC6695108; doi:10.1371/journal.pone.0220963)
Supplement: S2 Text — (DOCX) [file pone.0220963.s004.docx]

**S2 Text. Agro-vet semi-structured interview questions.**

Agrovet name: **________________________________________________________________**

Location: **_______________________________________________________________­­_____**

GPS coordinate: _______________________________________________________________

Closest survey village: **__________________________________________________________**

Distance to survey village (km):**___________________________________________________**

Visit date (dd/mm/yyyy): **________________________________________________________**

Enumerator names: **_____________________________________________________________**

Role in the agrovet shop:

□ Owner

□ Employee

| 1 | Do you currently have ND vaccine in stock? | | |
| --- | --- | --- | --- |
|  | | □Yes = 1  □ No = 2 | |
| 2 | What type of ND vaccines are in stock? *Check all that apply.* | | |
|  | | □ La Sota = 1  □ I-2 = 2  □ Other = 3 (*Describe) __________________________________________* | |
| 2a | Vaccine type (*Circle one)* La Sota I-2  Manufacturer:  Dose:  Price:  Usage instructions: | | |
| 2b | Vaccine type (*Circle one)* La Sota I-2  Manufacturer:  Dose:  Price:  Usage instructions: | | |
| 3 | Are there other poultry vaccines (not ND) available in the shop right now? Which ones? | | |
|  |  | | |
| 4 | From where do you get your vaccines? | | |
|  |  | | |
| 5 | Do you like to keep ND vaccines in stock at all times? | | |
|  | | □ Yes = 1  □ No = 2 | |
|  | *Why or why not?* | | |
| 6 | During how many days of the last year (from last August) did you have LA SOTA VACCINE available in your shop? *(estimate # of days out of 365)* | | |
|  | | ________ **#** days out of 365 | |
|  | *Notes:* | | |
| 7 | During how many days of the last year (from last August) did you have I-2 VACCINE available in your shop? *(estimate # of days out of 365)* | | |
|  | | ________ **#** days out of 365 | |
|  | *Notes:* | | |
| 8 | What are the pros and cons of La Sota vaccine? | | |
|  | **(+)** Pros | | **(-)** Cons |
| 9 | What are the pros and cons of I-2 vaccine? | | |
|  | **(+)** Pros | | **(-)** Cons |
| 10 | What do you consider when deciding which ND vaccines to stock? | | |
|  |  | | |
| 11 | What do your customers consider when buying ND vaccines? | | |
|  |  | | |
| 12 | What challenges exist for farmers using ND vaccines in your area? | | |
|  |  | | |
| 13 | What challenges exist for you as an agrovet with regards to stocking and selling ND vaccines? | | |
|  |  | | |
| 14 | How do you store your vaccines? | | |
|  |  | | |
| 15 | Select all the steps taken in this store | | |
|  | □ Refrigerator  □ Thermometer in fridge  □ Generator  □ Other (*describe)* ________________________________________ | | |
